# Transcriptomic Signature of Lipid Production in Australian *Aurantiochytrium* sp. TC20

**DOI:** 10.1007/s10126-025-10415-2

**Published:** 2025-02-06

**Authors:** Kim Jye Lee Chang, Eduardo Gorron Gomez, Esmaeil Ebrahimie, Manijeh Mohammadi Dehcheshmeh, Dion M. F. Frampton, Xue-Rong Zhou

**Affiliations:** 1 CSIRO Environment, P.O. Box 1538, Hobart, TAS 7001 Australia; 2 Genomics Research Platform, School of Agriculture, Biomedicine and Environment, La Trobe University, Melbourne, VIC Australia; 3 School of Animal and Veterinary Sciences, The University of Adelaide, Adelaide, SA 5371 Australia; 4 School of Biosciences, The University of Melbourne, Melbourne, VIC 3010 Australia; 5 CSIRO Agriculture and Food, P.O. Box 1700, Canberra, ACT 2601 Australia

**Keywords:** *Aurantiochytrium*, Climate change, Fatty acid, Marine aquaculture, Ocean carbon sink, Transcriptome

## Abstract

*Aurantiochytrium* not only excels in producing long-chain polyunsaturated fatty acids such as docosahexaenoic acid for humans, but it is also a source of essential fatty acids with minimal impacts on wild fisheries and is vital in the transfer of atmospheric carbon to oceanic carbon sinks and cycles. This study aims to unveil the systems biology of lipid production in the Australian *Aurantiochytrium* sp. TC20 by comparing the transcriptomic profiles under optimal growth conditions with increased fatty acid production from the early (Day 1) to late exponential growth phase (Day 3). Particular attention was paid to 227 manually annotated genes involved in lipid metabolism, such as *FAS* (fatty acid synthetase) and subunits of polyunsaturated fatty acids (*PUFA*) synthase. PCA analysis showed that differentially expressed genes, related to lipid metabolism, efficiently discriminated Day 3 samples from Day 1, highlighting the key robustness of the developed lipid-biosynthesis signature. Highly significant (pFDR < 0.01) upregulation of polyunsaturated fatty acid synthase subunit B (*PFAB*) involved in fatty acid synthesis, lipid droplet protein (*TLDP*) involved in TAG-synthesis, and phosphoglycerate mutase (*PGAM-2*) involved in glycolysis and gluconeogenesis were observed. KEGG enrichment analysis highlighted significant enrichment of the biosynthesis of unsaturated fatty acids (pFDR < 0.01) and carbon metabolism pathways (pFDR < 0.01). This study provides a comprehensive overview of the transcriptional landscape of Australian *Aurantiochytrium* sp. TC20 in the process of fatty acid production.

**Supplementary Information:**

The online version contains supplementary material available at 10.1007/s10126-025-10415-2.

## Introduction

Genus *Aurantiochytrium*, belonging to the family Thraustochytriaceae, has received considerable attention in the growing marine aquaculture and the nutraceuticals industry due to their high content of long-chain (≥ C20) polyunsaturated fatty acids (LC-PUFA), particularly docosahexaenoic acid (DHA) and eicosapentaenoic acid (EPA) (Sun et al. 2021; Raghukumar 2008). These are essential fatty acids for human health (Siriwardhana et al. 2012; Narayan et al. 2006), having remarkable benefits in the reduction of inflammation and risk of cardiovascular disease (Nestel et al. 2015; Jenkins et al. 2009). Wild fish stocks are currently the main source of omega-3 LC-PUFA in human nutrition (Lee et al. 2009; Sprague et al. 2016), yet this source has reached a limit. Microbial sources, including *Aurantiochytrium*, have gained greater importance as being both sustainable and scalable alternatives that moderate the pressure on these wild fish stocks (Moi et al. 2018; Lenihan-Geels and Bishop 2016).

*Aurantiochytrium* has additional environmental benefits, particularly in relation to marine ecosystem health and climate change. The genus, along with most Thraustochytrids, has the notable ability to grow on a range of carbon resources, such as agricultural waste, that offers a cost-effective and sustainable system for the production of long-chain polyunsaturated fatty acids (Li et al. 2015; Heggeset et al. 2019; Chen et al. 2022). Thraustochytrids also play an important role in contributing to marine carbon pools and the cycling and capture of atmospheric carbon dioxide (CO_2_) in marine systems (Sen et al. 2021).

Given the value of *Aurantiochytrium* in both environmental and industrial contexts, improving our understanding of the key underlying biosynthetic processes is of great interest. Lipid production and carbon metabolism are two of the processes that warrant further attention. There have been several reports on genome sequencing and transcriptome analysis of *Aurantiochytrium* sp. (Liu et al. 2016, 2020a; Ma et al. 2015; Prabhakaran et al. 2023; Heggeset et al. 2019; Morabito et al. 2020). In some of these previous studies, elicitors, such as low temperature, oxygen limitation, and nitrogen starvation (Bartosova et al. 2021; Chen et al. 2022; Heggeset et al. 2019; Ma et al. 2015; Song et al. 2022) have been employed to induce expression of genes involved in the production of fatty acids. These studies have each contributed to the current knowledge of genes involved in fatty acid biosynthesis, lipid metabolism, and energy production in *Aurantiochytrium* sp. Yet more information, and systems biology synthesis thereof, is needed in order to fully understand lipid production by these organisms.

A high level of biodiversity in thraustochytrids has been reported in Australia, particularly in the southeast coast of Tasmania (temperate) and the north of Queensland (tropical) (Lee Chang et al. 2012). Here, in a time-based study, we compared the transcriptomic profile of Australian *Aurantiochytrium* sp. TC20 at different points in lipid metabolism and biosynthesis, when fatty acid levels were higher (Day 3) against when fatty acids levels were lower (Day 1). A range of computational systems biology techniques were employed to understand the molecular basis of lipid metabolism and biosynthesis in this strain, as detailed below.

## Materials and Methods

### Strain and Cultivation

*Aurantiochytrium* strain TC 20 is deposited in the Australian National Algae Culture Collection (http://www.csiro.au/ANACC). Strain isolation information, medium preparation, and culturing conditions have been reported previously (Lee Chang et al. 2012). This study examined six cultures (triplicate flasks per time point) containing 1 L of culture medium in 2 L baffled flasks. The culture medium also included (percent *w*/*v*) sea salts (2), bacteriological peptone (0.2), and yeast extract (0.2) (all Sigma-Aldrich, St. Louis, MO, USA). Filter sterilized (0.2 µm) metal solution (1 mL/L) and vitamin solution (1 mL/L) were added after autoclaving. The peptone and yeast extract are considered to contain a complex source of amino acids (see “Discussion”). The metal solution contained (in milligrams per liter) MgSO_4_·7H_2_O (200), KH_2_PO_4_ (200), NaHCO_3_ (100), MnCl_2_·4H_2_O (9), Fe_3_Cl_3_·6H_2_O (3), ZnSO_4_·7H_2_O (1), CoSO_4_·5H_2_O (0.3), and CuSO_4_·5H_2_O (0.2). The vitamin mixture contained (in milligrams per liter) pyridoxine hydrochloride (0.2), thiamine (0.1), pantothenic acid (0.1), aminobenzoic acid (0.1), riboflavin (0.1), nicotinamide (0.1), biotin (0.04), folic acid (0.04), and vitamin B_12_ (0.002). All flasks were incubated in a shaking incubator at 20 °C and 200 rpm. Cultures were sampled on Day 1 and 3.

### Measurement of Fatty Acids in Aurantiochytrium sp. TC 20 Culture

Freeze-dried biomass (100 mg) was extracted using a modified version of Bligh and Dyer’s method, involving a mixture of dichloromethane (DCM), methanol (MeOH), and water (Bligh and Dyer 1959). The lipids were recovered in the lower DCM layer after phase separation. To enhance lipid recovery, a secondary extraction was conducted. Subsequently, solvents were evaporated *in vacuo*, and the gravimetric approach was used to determine lipid recovery.

A portion of the extracted lipids underwent transesterification using a mixture of methanol, dichloromethane, and HCl (10:1:1 v/v/v). This process converted complex lipids’ fatty acids into FAME, as outlined in a previous description (Lee-Chang et al. 2021). The individual fatty acids were then expressed as a percentage of the total fatty acids (TFA). For quantification of these fatty acids, gas chromatography (GC) was employed. The GC was conducted on an Agilent Technologies 7890A GC system equipped with a nonpolar Equity-1™ fused silica capillary column (15 m × 0.1 mm i.d., 0.1-mm film thickness), along with a flame ionization detector and split/splitless injector. The samples were introduced using the splitless mode at an initial oven temperature of 120 °C. Subsequently, the temperature was raised to 270 °C at a rate of 10 °C/min and then to 310 °C at 5 °C/min. Agilent Technologies ChemStation software was used for peak quantification.

To confirm the identity of individual components, gas chromatography-mass spectrometry (GC–MS) analysis of FAME was performed. This analysis took place on a Thermo Scientific 1310 GC system coupled with a TSQ triple quadrupole mass spectrometer. Samples were loaded via a Tripleplus RSH autosampler, and the analysis employed a non-polar HP-5 Ultra 2 bonded-phase column (50 m × 0.32 mm i.d. × 0.17 µm film thickness). The column’s polarity was similar to that of the GC analysis column. The analysis began at an initial oven temperature of 45 °C, held for 1 min, followed by a temperature ramp of 30 °C per minute up to 140 °C, and then at 3 °C per minute up to 310 °C, where it was held for 12 min. Helium (He) served as the carrier gas. The GC–MS operating parameters were as follows: electron impact energy 70 eV, emission current 250 µamp, transfer line 310 °C, source temperature 240 °C, scan rate 0.8 scan/s, and a mass range of m/z 40–650. Mass spectra were acquired and processed using Thermo Scientific XcaliburTM software (Waltham, MA, USA).

Most of the lipid content in thraustrochytrids (85–95%) consists of fatty acids, and therefore the total FAME was used as an indication of total lipid content. The biomass density (g biomass/L culture) was used to estimate the total FAME in mg/L culture.

### Transcriptome and Computational Systems Biology Analysis

RNA from three replicates for each day was extracted using Qiagen RNAeasy Plant Mini Kit (Qiagen, MD, USA), following the manufacture’s instruction and sequenced as previously described (Liang et al. 2018). Six total RNA samples belonging to two time points of Day 1 (*n* = 3) and Day 3 (*n* = 3) were sequenced by Illumina PE100 at Novogene (Hong Kong, China), with raw transcriptomic sequencing data in FASTQ format (paired reads, 100 bp). Transcriptomic analysis was performed using the QIAGEN CLC Genomic Workbench 20 tool as previously described (Govic et al. 2022; Heggeset et al. 2019). In short, quality testing and trimming of reads were performed based on the default settings (Trim Reads: Quality limit = 0.05, Maximum number of ambiguities = 2).

The annotated coding reference genome of *Aurantiochytrium* sp. T66 (GSE134374_T66_Genemodels, publicly available on Dec 19, 2019; https://www.ncbi.nlm.nih.gov/geo/query/acc.cgi?acc=GSE134374) was utilized as reference for read mapping and expression analysis. Reference genome construction and functional annotation were performed in 2019 based on de novo prediction of RNA-seq data (Heggeset et al. 2019). A second annotation was done against the genome of *Aurantiochytrium* sp. KH105 (publicly available on May 3, 2018; https://www.ncbi.nlm.nih.gov/datasets/genome/GCA_003116975.1/) but the annotation with the first genome showed higher mapping coverage (data not shown) and thus it was selected.

Cleaned (trimmed) reads were aligned to reference genome using CLC Genomic mapper, with the following parameters: mismatch cost = 2, insertion cost = 3, deletion cost = 3, length fraction = 0.6, and similarity fraction = 0.6. The reference genome of T66_GeneModels includes 11,683 coding genes, with 227 of these being manually annotated genes involved in lipid transport and metabolism (Heggeset et al. 2019). As the reference is a collection of 11,683 predicted coding genes, “One reference sequence per transcript” option in CLC Genomics Workbench for mapping was used to map the reads to de novo assembled gene models, as previously described (Heggeset et al. 2019).

During mapping, the maximum number of permitted hit matching for a read was set to 5 to inhibit extensive non-specific binding while avoiding underestimation of expression values. Differential expression analysis was performed to compare Day 3 against Day 1. Read counts (unique counts) were used for differential gene expression, and analysis was performed based on the Generalized Linear Model (GLM), an approach employed in edgeR (Robinson et al. 2010). The advantage of GLM is that curves are fitted to expression values without the assumption that the error on the values is normally distributed. Instead, the model assumption is that the read counts have a negative binomial distribution. Multivariate correction of *p*-values was performed with FDR statistics. Differentially expressed genes were selected based on FDR *p*-value < 0.05. Principle component analysis (PCA) of overall expression in all samples was employed for quality checking.

While statistical analysis was performed using read counts, visualization of expression data was carried out using RPKM (reads per kilobase of transcript per million mapped reads) values. Heatmaps were generated using the ClustVis webtool (Metsalu and Vilo 2015), with RPKM values being pre-processed for heatmap generation. At first, rows were centered, then unit variance scaling was applied to rows. Both rows and columns were clustered using correlation distance and average linkage methods.

Genes with significant differential expression were used as input for Gene ontology (GO) enrichment analysis in three terms of biological process (BP), molecular function (MF), and cellular component (CC) using STRING webtool, a database of predicted functional associations between proteins (Szklarczyk et al. 2023; Mering et al. 2003), and Comparative GO, a web application for comparative gene ontology and gene ontology-based gene (Fruzangohar et al. 2013).

Enrichment analysis and significance evaluation using the Fisher Exact test were performed using STRING to obtain a comprehensive view on molecular mechanisms where differentially expressed genes are involved. Enrichment analysis was performed against a range of databases including GO, KEGG, Pfam, and InterPro, as previously described (Alanazi and Ebrahimie 2016; Ebrahimie et al. 2015).

### Transcriptomic Signature of Discovery and Gene Selection by Multivariate Analysis

Multivariate analysis was employed to find the genes that can discriminate the samples with high and low contents of lipids. Analysis was performed using Minitab Statistical Software (www.minitab.com, Product version: 20.2). Selection of key genes in transcriptomic signature was performed based on the coefficient (weight) that each gene received in the separation of two groups (Day 1 samples from Day 3 samples) in Principal Component/Coordinates Analysis (PCoAAnalysis (PCA/PCoA) (Singh et al. 2018).

## Results

### Comparison of Fatty Acids Between Day 1 and Day 3 of Culture

In previous studies (Lee Chang et al. 2012, 2014), the growth curve and lipid profile of the Aurantiochytrium TC 20 strain, isolated from far north Queensland in Australia, were established and analyzed (Fig. S1). Based on these results, Days 1 and 3 were selected as sampling points, representing the start and the end of the linear growth phase and with the highest levels of DHA in % DW. During this period, the biomass increased ~ 2.4 times. Fatty acids profiles for Day 1 and Day 3 of Aurantiochytrium TC 20 culture were analyzed by GC and GC–MS (Table 1). Palmitic acid (16:0) was the most abundant saturated fatty acid, and its concentration decreases from Day 1 to Day 3. On the other hand, pentadecanoic acid (15:0) exhibited an increase from Day 1 to Day 3. This is consistent with previous findings associated with thraustochytrid growth in media containing branched amino acids (Morabito et al. 2020), whose catabolism can generate propionyl coA, which can be converted to pentadecanoic acid during fatty acid synthesis (Crown et al. 2015). In our case, the media contains yeast extract and peptone, both which contain a complex mix of amino acids. The increase of pentadecanoic acid suggests a key role of its catabolism during lipid synthesis.

**Table 1:** Biomass (g/L), fatty acid methyl esters (FAME) (mg/g dry biomass and mg/L culture) and fatty acid composition (% of total fatty acids and total DHA (g/L culture) of *Aurantiochytrium* TC 20. Stdv: standard deviation

	Day 1	Stdv	Day 3	Stdv
Biomass (g/L)	3.32	0.0519	7.88	0.06
Total FAME (mg/g)	214.26	0.5	264.91	0.9
Total FAME (mg/L)	0.71	0.01	2.09	0.03
Increase in FAMEs (fold vs Day 1)	1		2.94	
Fatty acid composition (% of total fatty acids)				
14:0	1.8	0.0	1.7	0.2
15:0	3.2	0.3	8.6	0.7
16:1ω9c	0.2	0.0	0.1	0.0
16:1ω7c	0.2	0.0	0.2	0.0
16:1ω7t	0.0	0.0	0.1	0.0
16:1ω5c	0.1	0.0	0.1	0.0
16:0	28.0	0.5	19.4	0.1
17:0	0.8	0.0	1.8	0.2
18:3ω6	0.1	0.0	0.1	0.0
18:4ω3	0.2	0.0	0.3	0.0
18:2ω6	0.3	0.0	1.0	0.2
18:3ω3	0.2	0.0	0.3	0.0
18:1ω9c	0.2	0.0	0.5	0.1
18:1ω7c	0.4	0.0	0.5	0.0
18:1a	0.0	0.0	0.0	0.0
18:0	0.9	0.0	0.7	0.1
20:4ω6	0.8	0.1	0.7	0.0
20:5ω3	0.3	0.0	0.4	0.0
20:3	0.2	0.0	0.2	0.0
20:3ω6	0.2	0.0	0.2	0.0
20:4ω3	0.6	0.0	0.7	0.0
20:0	0.2	0.0	0.2	0.0
22:5ω6	7.0	0.1	3.1	0.5
22:6ω3	51.8	0.5	56.3	0.8
22:4ω6	0.5	0.1	0.5	0.0
22:5ω3	0.4	0.4	0.8	0.0
Other FAs*	1.4	0.0	1.5	0.0
Total DHA (g/L)	0.37	0.01	1.18	0.02
Increase in FAMEs (fold Vs Day 1)	1		3.19	

*Other FAs include 18:1ω5c, 18:1ω9c, 20:1ω7c, C20 PUFA, 20:2ω6, C22PUFA, 22:1ω11c, 22:1ω9c, 22:1ω7c, 22:0

When the total FAMEs (in mg/L culture) are analyzed, an accumulation of FAMEs of 2.94-fold between Days 1 and 3 can be observed. The increase in docosahexaenoic acid (DHA) levels is relatively modest (< 5%). However, when the biomass increases and total FAME content is taken into account, the volumetric productivity of DHA showed a substantial increase of approximately 3.19-fold between Days 1 and 3, indicating ongoing DHA synthesis. This increase in DHA could potentially be attributed to both fatty acid synthase (FAS) and polyketide synthase (PKS) pathways, as both are involved in DHA synthesis in thraustochytrids (Morabito et al. 2019). However, the decrease of palmitic acid (16:0) and the moderate increase of pentadecanoic acid (15:0) might suggest that the FAS pathway is decreasing its activity, while the PKS pathway remains functioning. This gains additional support by the observation that most potential intermediates from the elongation of the main product of the FAS pathway, palmitic acid (fatty acids with 18 to 20 carbons) did not exhibit significant increases by Day 3, except for linoleic acid (18:2ω6). The only intermediate present at > 1% composition DW is docosapentaenoic acid (DPA) (22:5ω6) (Qiu 2003; Li et al. 2023). There are reports (Li et al. 2015) that DPA and DHA can be synthesized simultaneously by PKS, in a competitive fashion. Thus, the reduction in DPA can be explained as a compensation for a higher production of DHA under the PKS pathway.

### Quality of Sequencing Read and Expression Data

Quality control of NGS sequencing reads is presented in Tables S1 and S2 and Figs. S2–S9. Overall, the quality of sequencing data in all six samples was valid for further analysis with uniform length distribution, bell-shaped GC-content curve with a peak around 50%, great quality distribution with average PHRED score > 20, uniform and high coverage of base positions (up to position 100 bp), PHRED quality score bigger than 20 in positions of 1 bp to 100 bp of sequence reads. Samples were sequenced with high depth (32 ~ 52 million reads, with 3.24 ~ 5.25 Gb clean bases). This is crucial in working with non-model organisms where the sequence of reference genome and its annotation are not as good as the model organisms. The depth of sequencing was relatively uniform and high (in relation to the size of *Aurantiochytrium* sp. TC 20 genome): the mapping reached a coverage of > 15 million reads (~ 40% coverage).

**Fig. 1:**
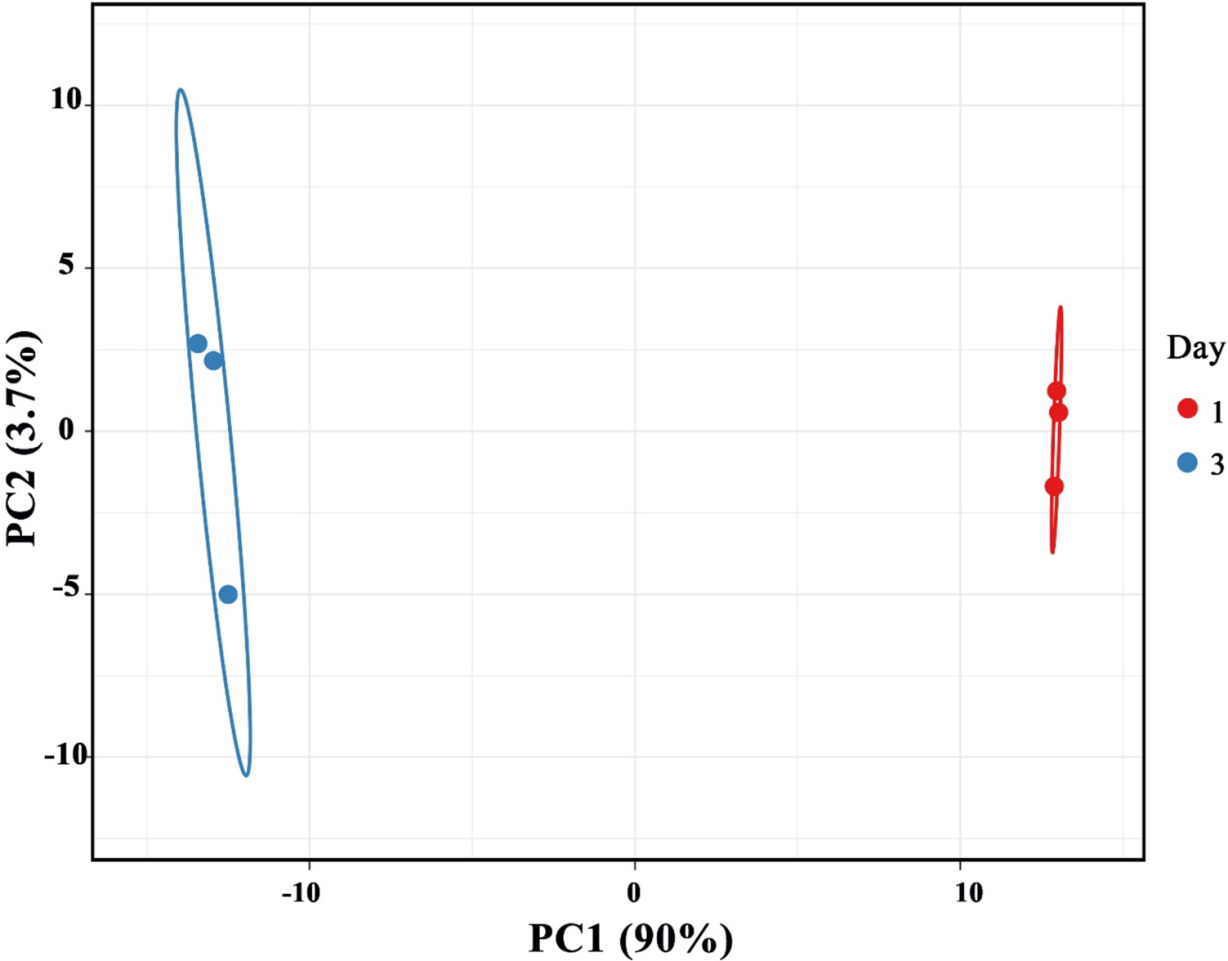
Principal component analysis applied to the three replicates for the differentially expressed genes (FDR *p*-value < 0.05) associated to lipid metabolism according to the annotation to *Aurantiochytrium* T66 genome

De novo assembled GSE134374_T66_Genemodels reference genome was used in this study. This reference includes 11,683 genes, derived from gene model analysis. One distinguishing benefit of this reference genome is that 227 genes are manually and closely annotated in lipid metabolism (Heggeset et al. 2019). The list of 11,683 genes in the reference genome and their FASTA sequences, as well as the list of 227 manually annotated genes in lipid metabolism, is presented in Supplementary Data 2. Functional groups for the 227 manually annotated genes are presented in Table 2. In summary, 10,651,277, 11,303,450, and 8,044,336 reads were mapped to GSE134374_T66_Genemodels reference genome for the replicate samples from Day 1. Day 3 replicates had 8,397,533, 8,063,296, and 5,202,947 mapped reads. Principle components analysis and heatmap analysis demonstrated the high quality of overall expression data, with Day 1 samples grouping together and showing clear separation (> 87% separation) from Day 3 samples (for an example see Fig. 1) (see Figs. S10–S12 for more details). KEGG enrichment analysis of upregulated genes and STRING analysis showed an enrichment of the biosynthesis of unsaturated fatty acids (pFDR < 0.01), carbon metabolism pathways (pFDR < 0.01), and pentose phosphate pathway (pFDR < 0.01), and Reactome pathways suggested a significant presence NAD(P)-binding domain superfamily (Tables S3 and S4). In contrast, other pathways related to carbohydrate metabolism and fatty acid degradation were not as significant (pFDR > 0.01).

**Table 2:** Manually annotated genes in *Aurantiochytrium* sp. TC20. reference genome (GSE134374_T66_Genemodels) that are involved in lipid metabolism*

Functional group	Number of genes in functional group
Associated reactions, cycles and shunts	22
Fatty acid synthesis	49
Glycerol uptake and conversion to glycerol phosphate	7
Glycolysis and gluconeogenesis	24
Lipases	19
PL-synthesis	27
PPP	7
TAG-synthesis	17
TCA	17
Transport in/out of mitochondria	11
β-oxidation	27
Total	227

*The complete list of 227 genes that are involved in lipid metabolism and their gene IDs are provided in Supplementary 2. The categories and annotated genes were first published in 2019 (Heggeset et al. 2019)

### Differential Expression Analysis for Day 1 vs Day 3 (Low vs High Lipid Production)

The overall number of differentially expressed genes between Day 1 and Day 3, with the lowest and highest lipid production, respectively, as well as for the category of 227 manually well annotated genes in lipid metabolism, is shown in Table 3. Interestingly, all 227 genes in lipid metabolism category showed differential expressions. Table S5 and Supplementary Material 2 provides the detailed differential gene expression information.

**Table 3:** Number of differentially expressed genes (FDR *p*-value < 0.05) in *Aurantiochytrium* sp. TC20 between Day 1 and Day 3 of culture

GSE134374_T66 reference genome	Differentially expressed genes	Upregulated genes	Downregulated genes
Overall (*n* = 11,683 genes)	5527	2800	2727
Manually well annotated genes in lipid metabolism (*n* = 227)	227	102	125

As the aim of this study is to find genes that are related to higher production and accumulation of fatty acids, the focus will be on the upregulated gene set (102 genes). The downregulated genes will be discussed in a further section.

### PUFA Synthesis Increase is Mostly Due to Increase in PKS Synthesis Pathway

**Fig. 2:**
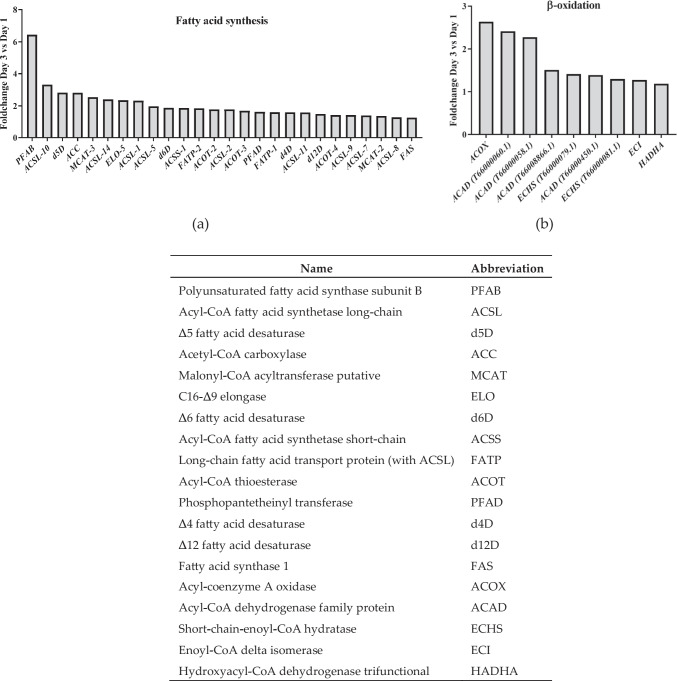
Main genes that showed upregulation on Day 3 compared to Day 1 and fold change. a Genes related to fatty acid synthesis. b Genes related to β-oxidation. The table shows the abbreviations used

The differential expression analysis of genes related to polyunsaturated fatty acid (PUFA) biosynthesis revealed significant upregulation of key genes, particularly within the PKS pathway, between Days 1 and 3 of culture (Fig. 2a). The subunit B of PUFA synthase (PFAB) exhibited the highest differential expression (> sixfold), indicating a substantial activation of the PKS pathway responsible for PUFA synthesis. This subunit contains multiple domains responsible for ketoacyl-ACP synthesis, chain length determination, enoyl-ACP reduction, and thioesterase activity (Guo et al. 2022). Given its role in elongating PUFA chains, its upregulation suggests a potential rate-limiting step in PUFA synthesis. In fact, the desaturase step has been mentioned as the limiting step for PUFA synthase in humans (Zhang et al. 2016). Also, since enoyl-ACP reductase uses NAPDH, it is expected that the upregulation of subunit B results in higher NADPH requirements.

Subunit C (PFAC) also exhibited upregulated gene expression on Day 3 but at a smaller rate (< twofold), and subunit A (PFAA) was not significantly upregulated on Day 3 compared to Day 1 (Fig. S13). In contrast, the FAS enzyme was only slightly upregulated (> twofold). Taken together with the lipid data and especially the decrease of palmitic acid (Table 1), this suggests an upregulation of PKS pathway, via PUFA synthase upregulation, and thus its predominant role in DHA synthesis over the FAS pathway towards Day 3.

FAS synthase also exhibited upregulation, albeit at a smaller magnitude (~ 1.5 fold) compared to PKS pathway enzymes. This pattern is in agreement with previous observations (Chen et al. 2020, 2022). Enzymes crucial for FAS-mediated elongation and desaturation processes, such as fatty acid synthetases and delta desaturases, also showed modest upregulation (2–threefold). In particular, delta 4 desaturase (d4D) is crucial to convert DPA (22:5w6, n-3) to DHA (Li et al. 2023). This enzyme is present predominantly in thraustochytrids (Qiu 2003). The decrease of palmitic acid levels on Day 3 (Table 1) might be explained by its elongation and desaturation of palmitic acid by these enzymes. A detailed comparison of gene expressions of *fas* vs *pfa* genes is presented in Fig. S13.

Surprisingly, several enzymes from the β-oxidation pathway were upregulated (Fig. 2b). The role of beta oxidation as an auxiliary pathway in microalgae remains unclear (Kong et al. 2018). An upregulation in β-oxidation enzymes was also observed by other authors (Deragon et al. 2021), who were unable to offer a definitive explanation but proposed that the upregulation of beta oxidation might be a metabolic response to the increased presence of fatty acids within the cells. Interestingly, our findings contradict those of another study (Chen et al. 2022), which reported a decrease in beta oxidation attributed to the downregulation of enoyl-CoA delta hydratase.

The acyl-CoA oxidase (ACOX) found in our work is similar to a peroxisomal ACOX in rats (Uniprot access # P07872) according to the annotation. The potential role of this enzyme as being specific for the metabolism of long chain fatty acids or its involvement in other metabolic pathways needs to be investigated.

### The Upregulation of Fatty Acid Synthesis Demands Higher Acetyl CoA and NADPH

**Fig. 3:**
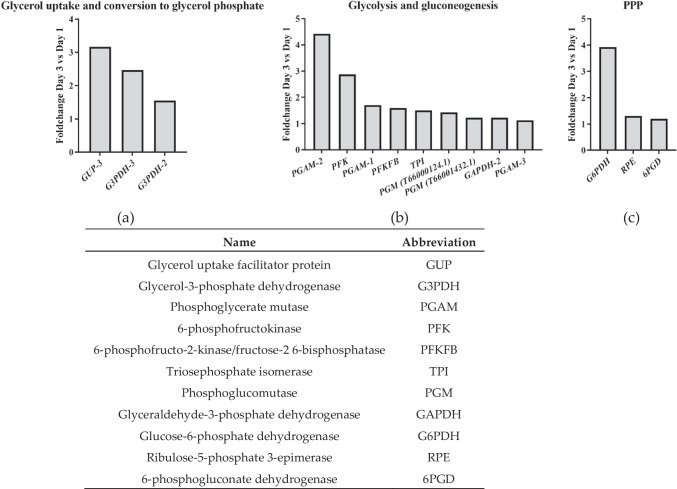
Main genes that showed upregulation on Day 3 compared to Day 1 and fold change. a Genes related to glycerol metabolism. b Genes related to glucose metabolism. c Genes related to pentose phosphate pathway (PPP). The table shows the abbreviations used

**Fig. 4:**
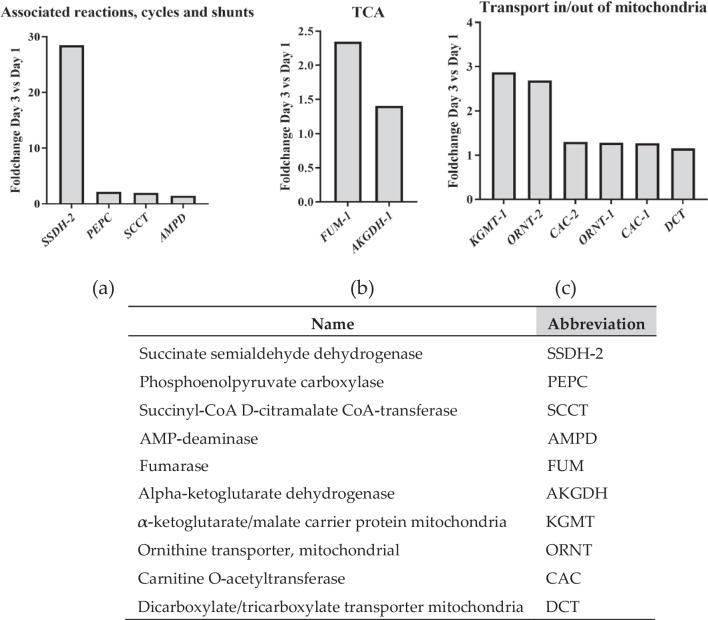
Main genes that showed upregulation on Day 3 compared to Day 1 and fold change. a Genes related to shunts associated to central metabolism. b Genes related to Krebs cycle (TCA). c Genes related to mitochondrial transport. The table shows the abbreviations used

Fatty acid synthesis in both PKS and FAS pathways needs two types of substrates: acetyl-CoA and NADPH. The observed upregulation of enzymes involved in glycolysis (phosphoglycerate mutase is remarkable with > fourfold upregulation) (Fig. 3a and b) plays a crucial role in maintaining an acetyl-CoA pool from carbohydrate catabolism (Xue et al. 2017b). The other substrate required, NADPH, would explain the observed upregulation of the pentose phosphate pathway (PPP) (Fig. 4c) (Li-Beisson et al. 2019), in agreement with previous studies (Heggeset et al. 2019). The enzyme glucose-6-phosphate dehydrogenase (G6PDH), the initial enzyme of the PPP and its primary bottleneck step, exhibited the highest upregulation (> threefold) in our results. Upregulation of G6PDH has been correlated with enhanced lipid accumulation in various microalgae (Xue et al. 2017a, 2020), with a reported increase in lipid yields by > 2 times. For example, the upregulation of G6PDH in *Schizochytrium* sp. H016 demonstrated a 1.9-fold increase in docosahexaenoic acid (DHA) yields (Feng et al. 2022), while a more modest increase of approximately 10% was observed in G6PDH-overexpressing *Aurantiochytrium* sp. SD116 (Cui et al. 2016). The upregulated expression of PGAM-2 could be also related to upregulation of PPP (Hitosugi et al. 2012). Overall, PPP upregulation would be supporting fatty acid synthesis.

However, our results point to a larger contribution of NADPH via a malate shuttle. Analysis of gene expression profiles revealed notable upregulation of four genes related to shunts (Fig. 4a). Among these, SSDH-2 exhibited the most significant increase of the entire data set, exceeding 28-fold in the 227-annotated genes. This finding is unexpected. SSDH-2 is recognized for its role in providing NADPH from derivates of amino acid metabolism, converting succinate semialdehyde to succinate, that can be converted to malate in the Krebs cycle in mitochondria. Malate can be exported to the cytoplasm and be used as a substrate for malic enzyme for additional NADPH production. Malic enzyme is a crucial source of NADPH in other oleaginous organisms (Alvarez Héctor et al. 2019; Dávila Costa et al. 2015). The elevated expression of SSDH-2 suggests a metabolic shift favoring amino acid catabolism for the production of Krebs cycle intermediates that might increase malate levels. This is possibly induced by nitrogen deprivation that starts at the last phase of the linear growth stage and stimulates lipid accumulation. Amino acid catabolism would also explain the increase in pentadecanoic acid (15:0) levels. Notably, such pronounced upregulation during the lipid accumulation phase mirrors findings in *Aurantiochytrium sp*. T66 (Heggeset et al. 2019). However, these authors did not discuss this phenomenon in detail.

Other three genes associated to shunts (Phosphoenolpyruvate carboxylase—PEPC -, Succinyl-CoA D-citramalate CoA-transferase – SCCT -, and AMP-deaminase – AMPD -) were upregulated, although at < threefold levels (Fig. 4a). PEPC and SSCT would help to provide intermediates of glycolysis and amino acid catabolism to the Krebs cycle such as PEP and succinyl-CoA (Friedmann et al. 2006). AMPD reduces AMP, downregulating the Krebs cycle at enzyme level, specifically in the step of conversion of isocitrate to alpha-ketoglutarate. This results in increased citrate levels, promoting the availability of acetyl-CoA for fatty acid synthesis (Chang et al. 2019; Lanaspa et al. 2012). Collectively, these interactions show a complex metabolic in thraustochytrids to optimize lipid production under nutrient stress conditions.

### Upregulation of Krebs Cycle and Associated Transporters Support NADPH Production from Amino Acid Catabolism

In our results, the transcript levels of two enzymes of the Krebs cycle, fumarase (FUM-1, the mitochondrial isoform) and alpha-ketoglutarate dehydrogenase, were increased at a significant level (Fig. 4b). The upregulation of both enzymes, and especially fumarase, could be crucial to increase the abundance of the key metabolite malate (Dao et al. 2022; Mühlroth et al. 2013). In addition to the malate shuttle described above, malate also serves as a precursor for acetyl-CoA (via malic enzyme), a substrate utilized by both fatty acid synthase (FAS) and polyunsaturated fatty acid synthase (PKS) pathways in the form of malonyl-CoA. The role of upregulation of the Krebs cycle to support cell growth cannot be excluded.

The upregulation of mitochondrial transporters identified in this study (Fig. 4c) can be explained by their connections to the metabolic pathways previously described. The enzyme α-ketoglutarate/malate carrier protein mitochondria is connected to the Krebs cycle (Dao et al. 2022). Carnitine O-acetyltransferase (CAC) enzymes play pivotal roles in fatty acid transport processes within the mitochondria. The ornithine transporter (ORNT) upregulation could be linked to enhanced amino acid degradation, particularly prominent under conditions of nitrogen deprivation. This phenomenon is consistent with previous studies (Liang et al. 2019; Prihoda et al. 2012) and suggests a response mechanism to nitrogen scarcity. Moreover, the involvement of intermediates from the urea cycle in signalling pathways related to nitrogen starvation in algae further stresses the significance of ORNT upregulation (Monteiro et al. 2023). The dicarboxylate/tricarboxylate transporter (DCT) functions as a carrier of intermediates essential for fatty acid synthesis in a role analogue to the malate shuttle (Dolce et al. 2014).

### Lipid Synthesis is Accompanied by Several Events of Lipid Translocation

**Fig. 5:**
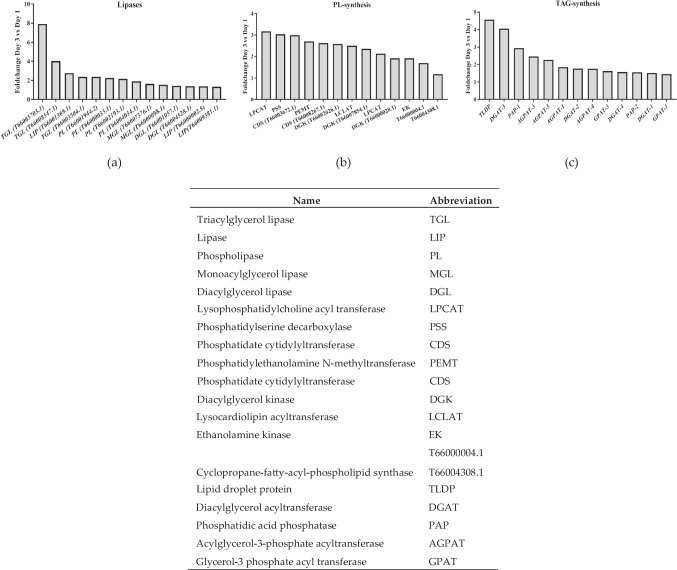
Main genes that showed upregulation on Day 3 compared to Day 1 and fold change. a Genes related to lipases. b Genes related to phospholipid (PL) synthesis. c Genes related to triacylglycerol (TAG) synthesis. The table shows the abbreviations used

Several enzymes involved in lipid metabolism, including lipases and enzymes related to phospholipid and triacylglycerol (TAG) synthesis were upregulated on Day 3 (Fig. 5). They play crucial roles in providing intermediates for PUFA synthesis (Dums et al. 2018; Janssen et al. 2019; Khozin-Goldberg et al. 2005; Xue et al. 2017b) by means of translocation of membrane lipids to storage lipids. For example, lysophosphatidylcholine acyltransferase (LPCAT) facilitates lipid pool exchange (Połońska et al. 2021) and provides substrates for desaturase-like enzymes during PUFA synthesis (Hashidate-Yoshida et al. 2015). Similarly, enzymes such as phosphatidate cytidylyltransferase, also known as CDP-diacylglycerol synthase (CDS), cyclopropane-fatty-acyl-phospholipid synthase, and ethanolamine kinase (ETK) play key roles in this process (Bai et al. 2019; Ye et al. 2024; Zhang et al. 2021). Notably, the marked increase in lipid droplet protein (LDP) expression, with a fold change of ~ 4.5-fold, features its significance in promoting TAG accumulation in lipid bodies, particularly favoring unsaturated fatty acids (Li-Beisson et al. 2019; Siegler et al. 2017). These enzymes are also involved in the construction of cell membranes, and thus their role in membrane synthesis necessary for cell duplication cannot be excluded.

### Downregulated Genes

The main focus of this study was the identification of upregulated genes, since they would be more promising targets for future biotechnological improvements regarding PUFA production. However, to have a full picture of the transcriptomic changes between Days 1 and 3, the downregulated genes will be briefly discussed. From the 125 genes that were part of the 227 annotated genes and identified as downregulated (Table S6), 57 had a > twofold change and those are the ones considered for discussion. Only eight genes have > fourfold change in expression. The gene with the highest downregulation was glutamate decarboxylase (GAD) (> sevenfold). GAD is a key enzyme in the GABA shunt, a diversion from the Krebs cycle at the level of succinyl-CoA (Feehily et al. 2013; Shelp et al. 2012). The enzyme aspartate aminotransferase (AST), which is also related to this pathway (Han et al. 2021), shows also > fivefold downregulation. Several of the other highly downregulated genes are related to the Krebs cycle (Yan and Wang 2023), in contrast to fumarase (FUM-1) and alpha-ketoglutarate dehydrogenase, which were upregulated. This suggests a finely tuned regulation to increase malate concentrations, which may contribute to lipid accumulation. In particular, the downregulated isoform of fumarase is FUM-2, which is a cytosolic isoform, and it is not an essential gene. It is involved in fumarate accumulation in cytosol in Arabidopsis and its knockout accumulates malate (Pracharoenwattana et al. 2010), which further points out to the role of malate during lipid synthesis.

The enzyme ribose-5-phosphate isomerase was < fourfold downregulated. This is the first enzyme of the non-oxidative branch of the pentose phosphate pathway that directs the sugars towards nucleotide synthesis. Therefore, its downregulation favors the recycling of phosphate sugars to the oxidative branch to sustain NADPH generation (Heintze et al. 2016; Wamelink et al. 2010). The downregulation of other enzymes such as enolase (< sixfold), transketolase (< twofold) and phosphoglycerate kinase (< threefold) is in agreement with this. Finally, among the downregulated genes, there are isoforms of few enzymes related to fatty acid synthesis and beta oxidation. It has been established that there are several isoforms of these enzymes with specific functions (Batsale et al. 2023; Carman and Han 2019) that are yet to be clearly established in thraustochytrids.

## Discussion

Research into the pathways involved in fatty acid synthesis in thraustochytrids remains a priority. Despite advancements in understanding the properties of specific enzymes like polyketide synthase (PKS), fundamental questions persist regarding the existence of multiple pathways for PUFA synthesis and their interconnections. This study offers valuable insights into these pathways and their relationship with central metabolism.

In contrast to previous transcriptomic approaches utilizing elicitors (Chen et al. 2022; Ma et al. 2015; Heggeset et al. 2019), our study employed a time-based transcriptomic profiling strategy to delineate changes in lipid production in *Aurantiochytrium*. By focusing on key growth cycle stages without the influence of elicitor-responding genes, we achieved a clean and specific transcriptomic signature of lipid production changes. This approach enhances the reliability and specificity of our findings, providing a clearer understanding of the transcriptional landscape associated with lipid metabolism alterations in *Aurantiochytrium*. By elucidating these dynamics, our study contributes to a more comprehensive understanding of lipid biosynthesis regulation in thraustochytrids.

Quality control measures were rigorously implemented to ensure the reliability of the next-generation sequencing (NGS) data in this study, as detailed in Supplementary 1. The sequencing data from all six samples exhibited high quality, characterized by uniform length distribution, a bell-shaped GC-content curve with a peak around 50%, an average PHRED score greater than 20, and uniform and high coverage of base positions, indicating robust sequencing depth. Such meticulous quality control is particularly crucial when working with non-model organisms like *Aurantiochytrium* sp., where reference genome sequences and annotations may be less comprehensive compared to model organisms. Moreover, the utilization of a de novo assembled reference genome, comprising 11,683 genes, further enhanced the reliability of our analysis. This reference genome includes 227 genes that are manually and closely annotated to lipid metabolism, highlighting the robustness of our genomic analysis (Heggeset et al. 2019).

The upregulation of PKS genes surpassed that of fatty acid synthase (FAS) genes, indicating higher PKS-related activity compared to FAS in this organism under the conditions tested. This trend aligns with findings reported by multiple researchers in previous studies. The elevated expression levels of PKS relative to FAS support the hypothesis that PKS serves as the primary route for DHA synthesis in thraustochytrids over FAS.

Both pathways require two components: a large NADPH pool and acetyl-CoA (Bartosova et al. 2021). Given the central importance of NADPH, can boosting the activity of the pentose phosphate pathway (PPP) effectively enhance lipid biosynthesis in thraustochytrids? Recent research demonstrates enhanced lipid accumulation when glucose-6-phosphate dehydrogenase (G6PDH) is upregulated. Notably, G6PDH upregulation has correlated with > twofold lipid accumulation in different microalgae (Xue et al. 2017a, 2020). In line with these findings, upregulation of G6PDH in *Schizochytrium* sp. H016 also showed an increase in DHA yields by 1.9 times (Feng et al. 2022). The increase was more modest in G6PDH-overexpressing *Aurantiochytrium* sp. SD116 (~ 10%) (Cui et al. 2016). These results are similar to the ones reported by other authors (Heggeset et al. 2019). On the other hand, the downregulation of ribose−5-phosphate isomerase has not been reported in thraustochytrids and deserves further investigation.

The interplay between the pentose phosphate pathway (PPP), glycolysis, and amino acid metabolism determines NADPH availability for lipid biosynthesis. While the PPP is a primary source of NADPH, competition with glycolysis for glucose-derived substrates may limit its effectiveness in supplying NADPH for lipid accumulation, and thus NADPH would become a critically limiting substrate. This can be compensated by the upregulation of genes involved in amino acid metabolism, such as observed with SSDH, particularly elevated during the lipid accumulation phase. This finds support in previous studies where enhancing NADPH supply, either through inositol supplementation or the manipulation of NADP-malic enzyme activity, led to notable increases in lipid content (Liu et al. 2019). SSDH upregulation has been reported in other studies of *Aurantiochytrium* sp. T66 (Heggeset et al. 2019). However, these authors did not discuss in depth this finding.

There have been other lines of evidence for the key role of amino acid catabolism during the process of lipogenesis, as suggested by observations in adipocytes (Green et al. 2016; Torres et al. 2023). A nitrogen-rich media proved to be more effective for DHA accumulation compared to glucose-rich media in *Thraustochytrium* sp. RT2316-16 (Valdebenito et al. 2022); the amino acid composition within the growth media has an impact on DHA accumulation. An in silico analysis of the metabolome of *Thraustochytrium* RT2316-16 reveals a substantial allocation of metabolic reactions dedicated to both lipid and amino acid metabolism (463 metabolic steps each), almost doubling those devoted to carbohydrate metabolism (247 steps) (Shene et al. 2024). These authors showed that glucose by itself could not explain the changes in biomass in this organism and that the amino acid composition of the media affected the lipid profile and content of the strain. The role of amino acid catabolism can explain the observed increase in pentadecanoic acid and the upregulation of genes associated with key steps of the Krebs cycle, the urea cycle, PEPC and SCCT in our study. Moreover, the absence of upregulated amino acid synthesis genes in this study supports the notion that amino acid catabolism plays a central role during the lipid accumulation stage in *Aurantiochytrium*.

The upregulation of genes associated with lipid mobilization from phospholipid pools and triacylglycerols highlights the dynamic nature of lipid metabolism in thraustochytrids. However, the implications of the observed increase in genes related to β-oxidation remain unclear. The potential role of the peroxisome in this process cannot be excluded. The acyl-CoA oxidase (ACOX) in our study was annotated as localized in the peroxisome. Notably, the *Aurantiochytrium* genome contains three ACOX genes, all featuring peroxisomal signal peptides (Watanabe et al. 2021). CRISPR-knockout experiments targeting two of these three variants resulted in reduced lipid accumulation, although this effect was not observed when ACOX3 was rendered non-functional. Another potential explanation would be related to amino acid catabolism. Enzymes from β-oxidation, particularly ACOX and enoyl-CoA hydratase, have been reported to be involved in the catabolism of branched amino acids towards propionyl-CoA and acetyl-CoA (Hildebrandt et al. 2015). This would explain both the upregulation of some β-oxidation enzymes and the presence of pentadecanoic acid.

Taken together, our findings align with the notion that thraustochytrids prefer to the polyketide synthase (PKS) pathway over the fatty acid synthase (FAS) pathway for PUFA synthesis. This preference is supported by the upregulation of genes associated with the PKS pathway, suggesting a concerted effort to enhance PUFA biosynthesis. Furthermore, while the increase in amino acid catabolism enzymes has been documented in other organisms, this study represents one of the first reports of such a phenomenon in thraustochytrids. Our observations suggest a potential metabolic shift towards amino acid catabolism to obtain NADPH via the malate shuttle during the lipid accumulation stage. Further studies are needed to elucidate this relationship between lipid and amino acid metabolism for enhancing lipid production in these microorganisms.

While our transcriptomic analysis sheds light on gene expression changes underlying lipid metabolism in thraustochytrids, a comprehensive understanding requires proteomic and metabolomic analyses. For instance, a metabolic profiling for *Aurantiochytrium* T66 (Bartosova et al. 2021) reported the decrease of all amino acids during the lipid accumulation stage while most TCA intermediates remain unchanged. However, these authors employed minimal media with ammonium as a nitrogen source. In their results, the concentration of palmitic acid was higher than that of DHA, which is contrary to what we found. Other authors (Perez et al. 2019; Yang et al. 2020; Liu et al. 2020b; Pei et al. 2017) have conflicting results about the increase of Krebs cycle and glycolysis metabolic intermediates, not always correlated with the transcriptomic data. These studies are difficult to compare since they employ different media and growth conditions, which have been shown to affect DHA metabolism and accumulation in thraustochytrids. Thus, future studies should compare the metabolome and proteome to provide a deeper insight into lipid metabolism during the lipid accumulation phase.

Biological and environmental treatments have been suggested to promote the expression of genes and pathways involved in omega-3 fatty acid production in *Aurantiochytrium* (Chauhan et al. 2023; Heggeset et al. 2019; Han et al. 2020). The key genes discovered herein could be employed for promoter analysis and discovery of key regulators, such as transcription factors, involved in *Aurantiochytrium* sp. TC20 lipid production, potentially leading to further enhancement of DHA production. A clear example would be SSDH-2 regulatory elements, which deserve to be studied in more detail and could become a promising target for future metabolic engineering.

Enhancing lipid production in thraustochytrids holds significant promise for both human health and environmental sustainability. Thraustochytrids play a crucial role in marine ecosystems by sequestering carbon and converting it into biomass and lipid compounds, contributing to the carbon sink role of world oceans (Gruber et al. 2023; Heinze et al. 2015). In fact, thraustochytrids could represent one of the largest pools of carbon in the ocean (Sen et al. 2021). Moreover, thraustochytrids, such as the Australian native strain of *Aurantiochytrium* examined in this study, offer a sustainable alternative for the production of omega-3 long-chain polyunsaturated fatty acids (PUFAs), reducing our dependence on animal-derived sources and wild fish populations (Patel et al. 2021). This research highlights the potential of thraustochytrids, particularly indigenous strains, for future applications in DHA production, offering promising avenues for sustainable resource utilization and ecosystem preservation.

## Supplementary Information

Below is the link to the electronic supplementary material.

Supplementary file1 (DOCX 1426 KB)

Supplementary file2 (XLSX 1904 KB)

## Data Availability

No datasets were generated or analysed during the current study.
