# Supplementary material for: Transcriptomic Signature of Lipid Production in Australian Aurantiochytrium sp. TC20
Source: Mar Biotechnol (NY). 2025 Feb 6;27(1):43. doi: 10.1007/s10126-025-10415-2 (PMC11802676; doi:10.1007/s10126-025-10415-2)
Supplement: Supplementary file 1 — Supplementary file1 (DOCX 1426 KB) [file 10126_2025_10415_MOESM1_ESM.docx]

Supplementary Material

Transcriptomic signature of lipid production in Australian *Aurantiochytrium* sp. TC20

Kim Lee Chang ^1^, Eduardo Gorron^1*^, Esmaeil Ebrahimie^2,3,4^, Manijeh Mohammadi Dehcheshmeh^2^, Dion M. F. Frampton^1^ and Xue-Rong Zhou ^5^

|  |
| --- |

^1^ CSIRO Environment P.O. Box 1538, Hobart, TAS 7001, Australia; [Kim.Leechang@csiro.au](mailto:Kim.Leechang@csiro.au), Dion.Frampton@csiro.au

^2^ Genomics Research Platform, School of Agriculture, Biomedicine and Environment, La Trobe University, Melbourne, Victoria, Australia; E.Ebrahimie@latrobe.edu.au

^3^ School of Animal and Veterinary Sciences, The University of Adelaide, Adelaide, SA 5371, Australia; esmaeil.ebrahimie@adelaide.edu.au

^4^ School of Biosciences, The University of Melbourne, Melbourne, VIC 3010, Australia

^5^ CSIRO Agriculture and Food, P.O. Box 1700, Canberra, ACT 2601, Australia; xue-rong.zhou@csiro.au

***** Corresponding author:

[e.gorrongomez@csiro.au](mailto:e.gorrongomez@csiro.au)

Phone: +61-1300-363-400

**Figure S1.** Growth curve of Aurantiochytrium sp. TC20 grown in 2% glucose medium during 21 days, along with total lipid and DHA volumetric productivities for selected timepoints. All values provided in g/L of culture.

**Table S1**. Sequencing data production

| Sample | Raw reads | Clean reads | Clean bases | Error rate% | Q20(%) | Q30(%) | GC content (%) |
| --- | --- | --- | --- | --- | --- | --- | --- |
| D1_1 | 47584503 | 47485814 | 4.8G | 0.04 | 96.17 | 85.71 | 52.99 |
| D1_2 | 52299866 | 51137745 | 5.16G | 0.04 | 96.14 | 85.71 | 52.75 |
| D1_3 | 36117049 | 36066837 | 3.64G | 0.04 | 96.19 | 85.74 | 52.86 |
| D3_1 | 52148008 | 51962712 | 5.25G | 0.04 | 96.33 | 86.11 | 52.19 |
| D3_2 | 50363727 | 50154224 | 5.07G | 0.04 | 96.34 | 86.15 | 52.17 |
| D3_3 | 32106530 | 32037869 | 3.24G | 0.04 | 96.46 | 86.40 | 52.15 |

**Table S2**. Sequencing data production

| Sample_name | D1_1 | D1_2 | D1_3 | D3_1 | D3_2 | D3_3 |
| --- | --- | --- | --- | --- | --- | --- |
| Total reads | 47485814 | 51137745 | 36066837 | 51962712 | 50154224 | 32037869 |
| Total mapped | 43094473 (90.75%) | 46099167 (90.15%) | 32632707 (90.48%) | 46582860 (89.65%) | 44882840 (89.49%) | 28764533 (89.78%) |
| Multiple mapped | 304219 (0.64%) | 322977 (0.63%) | 229748 (0.64%) | 627981 (1.21%) | 610274 (1.22%) | 414687 (1.29%) |
| Uniquely mapped | 42790254 (90.11%) | 45776190 (89.52%) | 32402959 (89.84%) | 45954879 (88.44%) | 44272566 (88.27%) | 28349846 (88.49%) |
| Reads map to '+' | 21382989 (45.03%) | 22884123 (44.75%) | 16185870 (44.88%) | 23034000 (44.33%) | 22186576 (44.24%) | 14209177 (44.35%) |
| Reads map to '-' | 21407265 (45.08%) | 22892067 (44.77%) | 16217089 (44.96%) | 22920879 (44.11%) | 22085990 (44.04%) | 14140669 (44.14%) |
| Non-splice reads | 41146356 (86.65%) | 43963682 (85.97%) | 31171151 (86.43%) | 45064884 (86.73%) | 43390947 (86.52%) | 27812636 (86.81%) |
| Splice reads | 1643898 (3.46%) | 1812508 (3.54%) | 1231808 (3.42%) | 889995 (1.71%) | 881619 (1.76%) | 537210 (1.68%) |

Total reads, number of total filtered reads (Clean data). Total mapped, total number of reads that could be mapped to the genome. Multiple mapped, number of reads that could be mapped to multiple sites in the reference genome. Uniquely mapped, number of reads that can be uniquely mapped to the reference genome. Reads map to '+' and reads map to '-', number of reads that mapped to positive strand or minus strand, respectively. Splice reads, number of reads that could be segmented and mapped to 2 exons. Non-splice reads, number of reads that can be mapped entirely to a single exon.


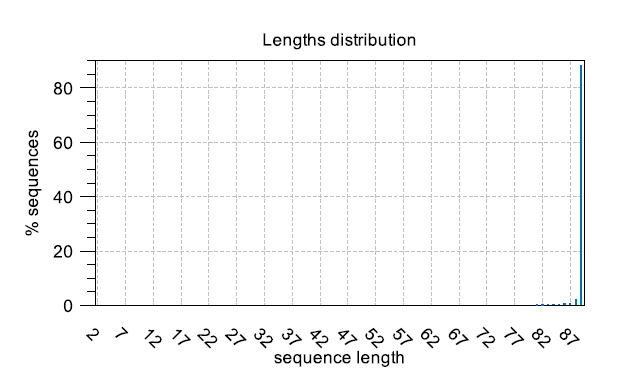


**Figure S2.** Distribution of sequence length. X-axis, sequence length in base pairs; Y-axis, number of sequences featuring a particular length normalised to the total number of reads.


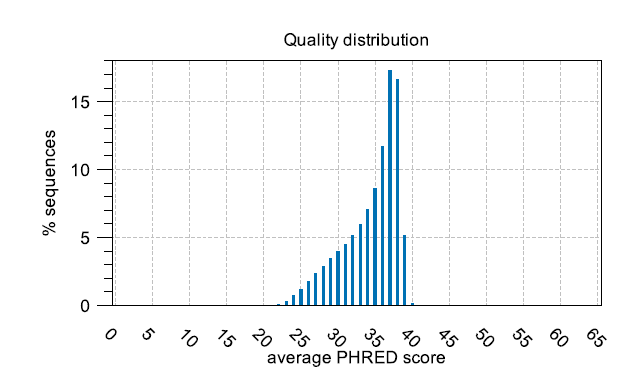


**Figure S3.** Distribution of average sequence quality scores. X-axis, PHRED score; Y-axis, number of sequences observed as the quality score normalised to the total number of reads.


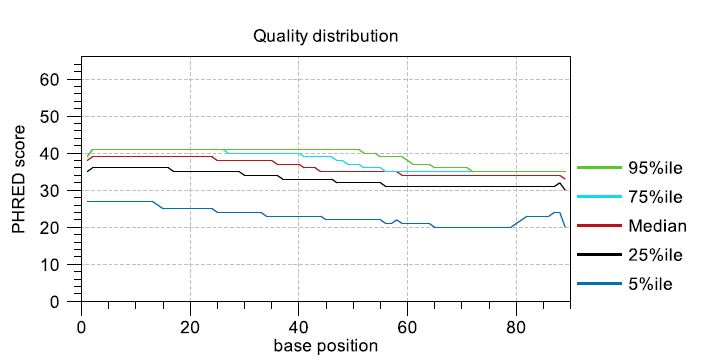


**Figure S4.** Base quality distribution along the base positions. X-axis, base position; Y-axis, median and percentiles of quality scores observed at that base position.


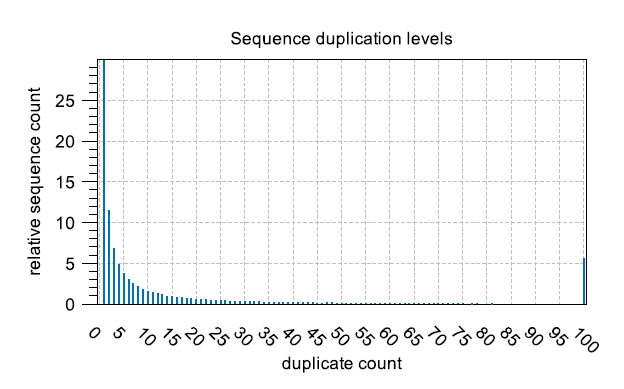


**Figure S5.** Duplication level distribution along the base positions. Duplication levels are the count of how often a particular sequence has been found. X-axis, duplicate count; Y-axis, number of sequences that have been found that many times normalised to the total number of reads.


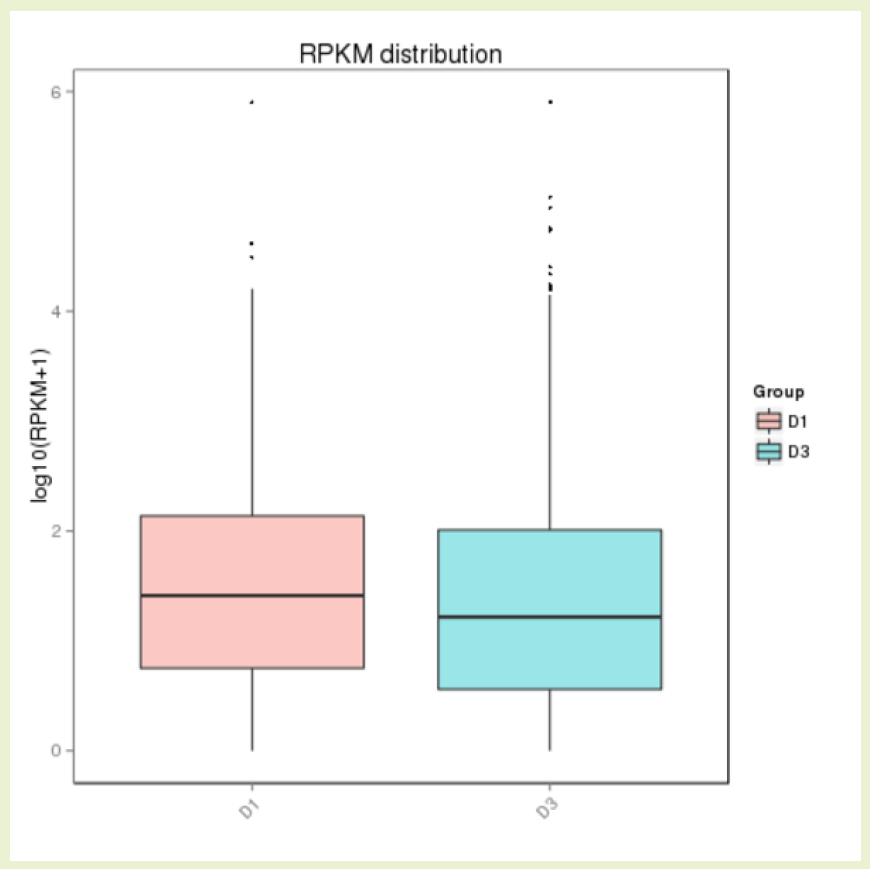


**Figure S6.** RPKM distribution along the base positions. X-axis, sample groups; Y-axis, the log_10_(RPKM+1).


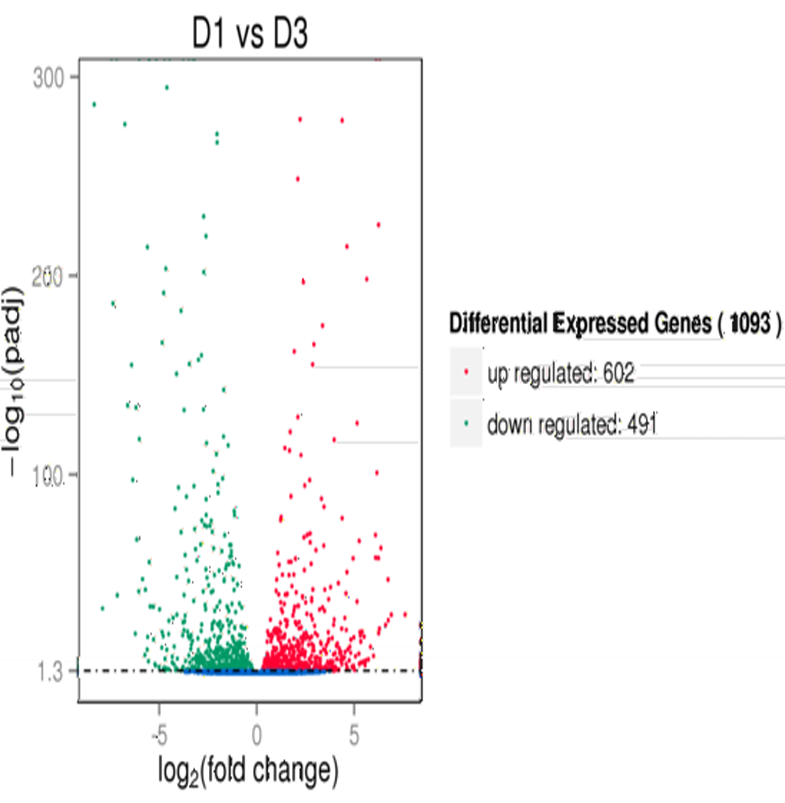


**Figure S7:** Volcano plot for all the genes analysed in this study**.** From a total of 11683 genes (see Supplementary Data 3), 1093 showed differential expression, including the 277 genes associated to lipid metabolism. Difference genes with statically significance were represented by red dots (upregulated) and green dots (downregulated).


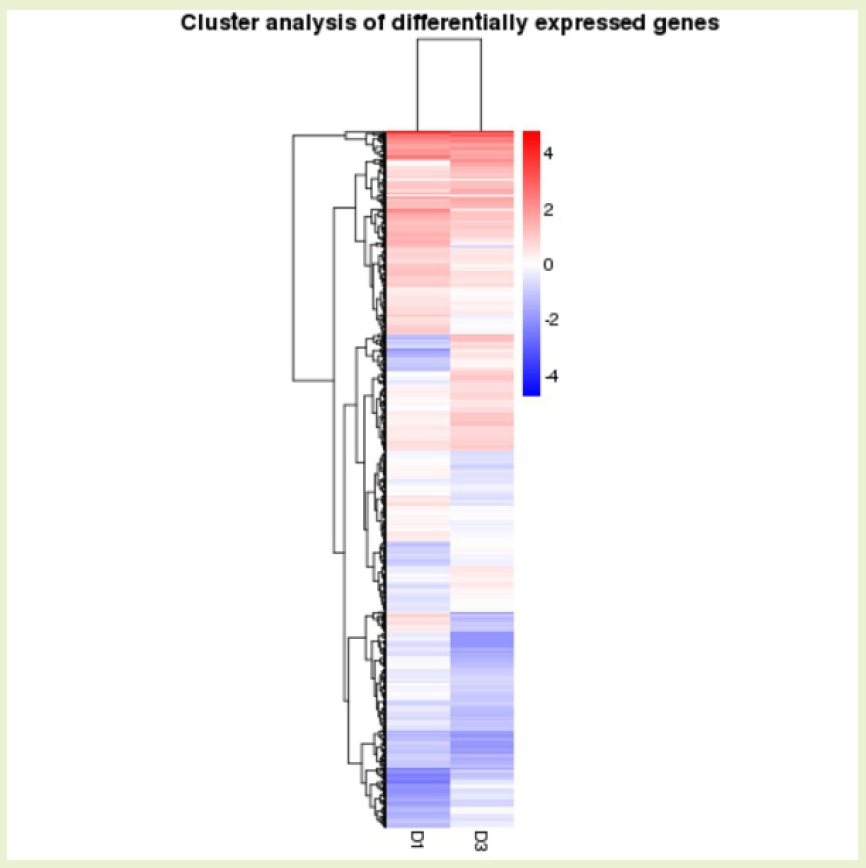


**Figure S8.** Cluster analysis of differentially expressed genes, withthe overall RPKM from all replicates clustered by log_10_(RPKM+1) value. Red represents genes with high expression level, blue represents genes with low expression level.


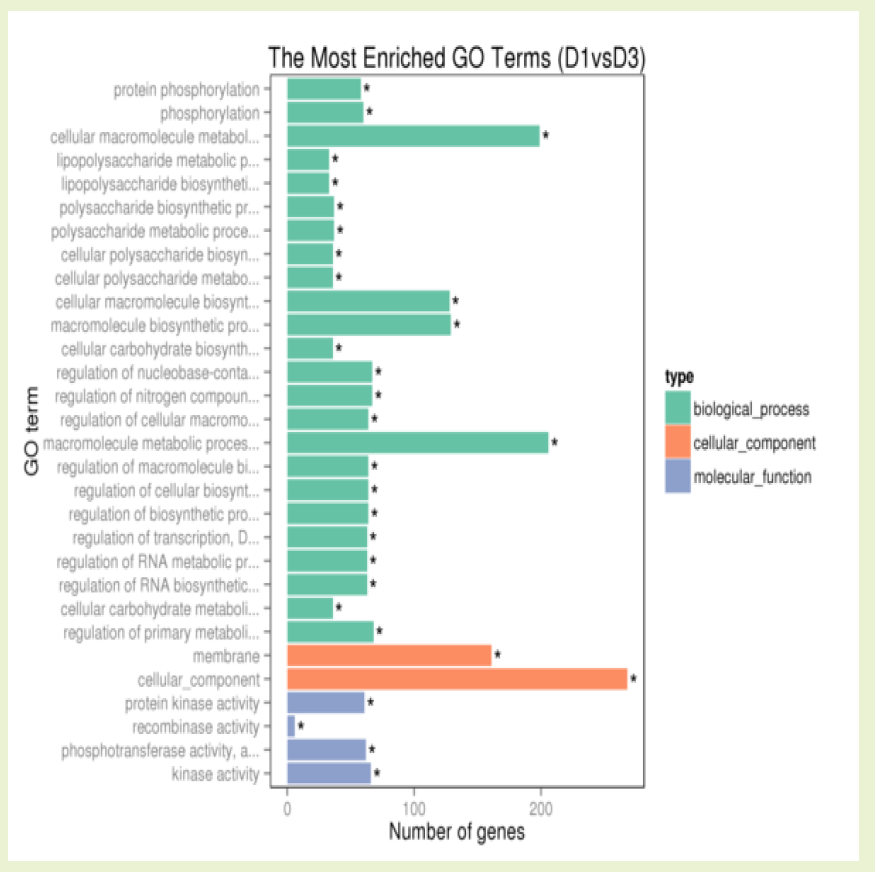


**Figure S9.** Gene ontology enrichment of differentially expressed genes.


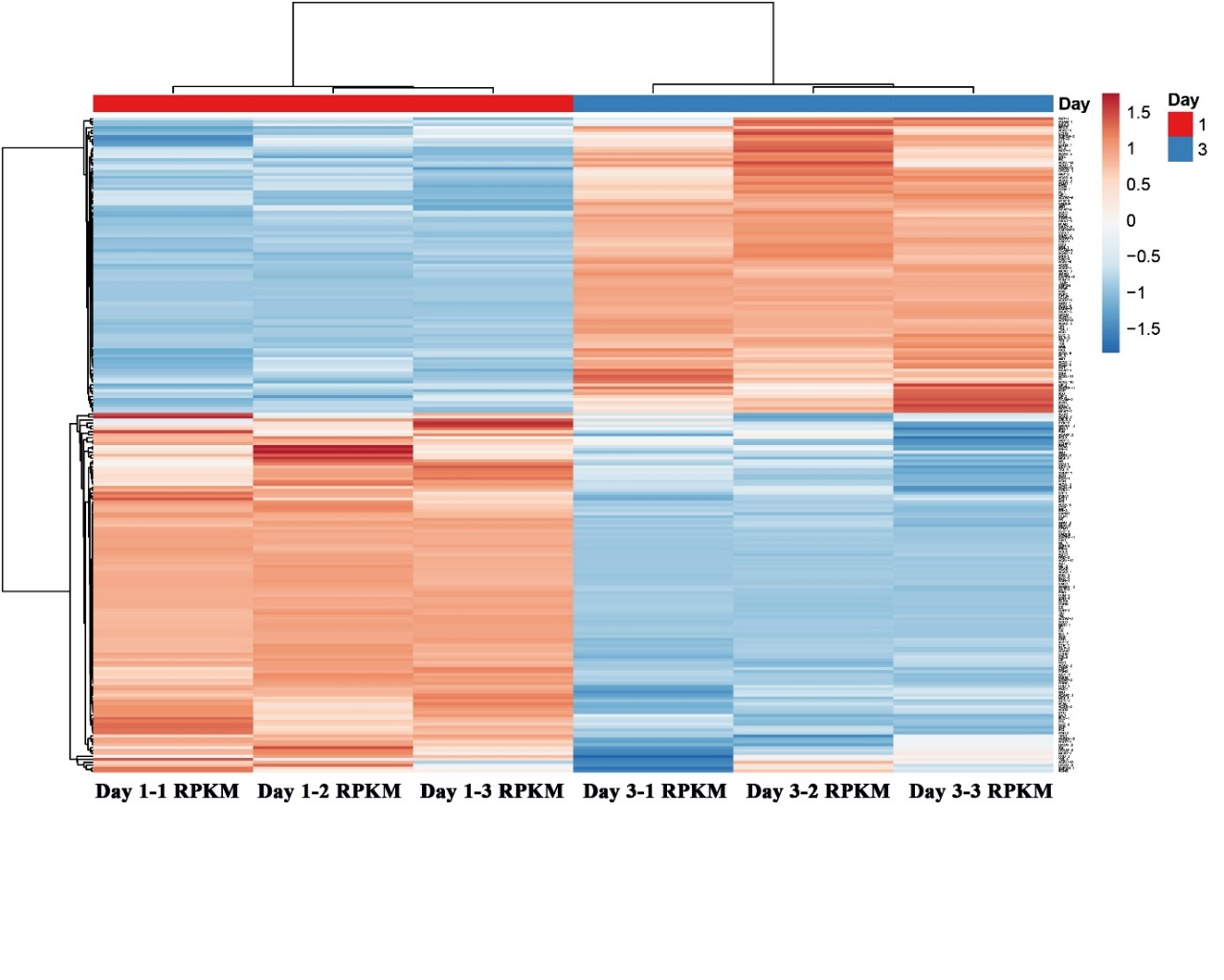


**Figure S10.** Heatmap of differentially expressed genes (FDR p-value<0.05) in lipid metabolism. RPKM values were pre-processed for heatmap generation. Rows were centered, then unit variance scaling was applied to rows. Both rows and columns were clustered using correlation distance and average linkage methods. Rows represent genes (n=227), and columns represent samples (n=6).


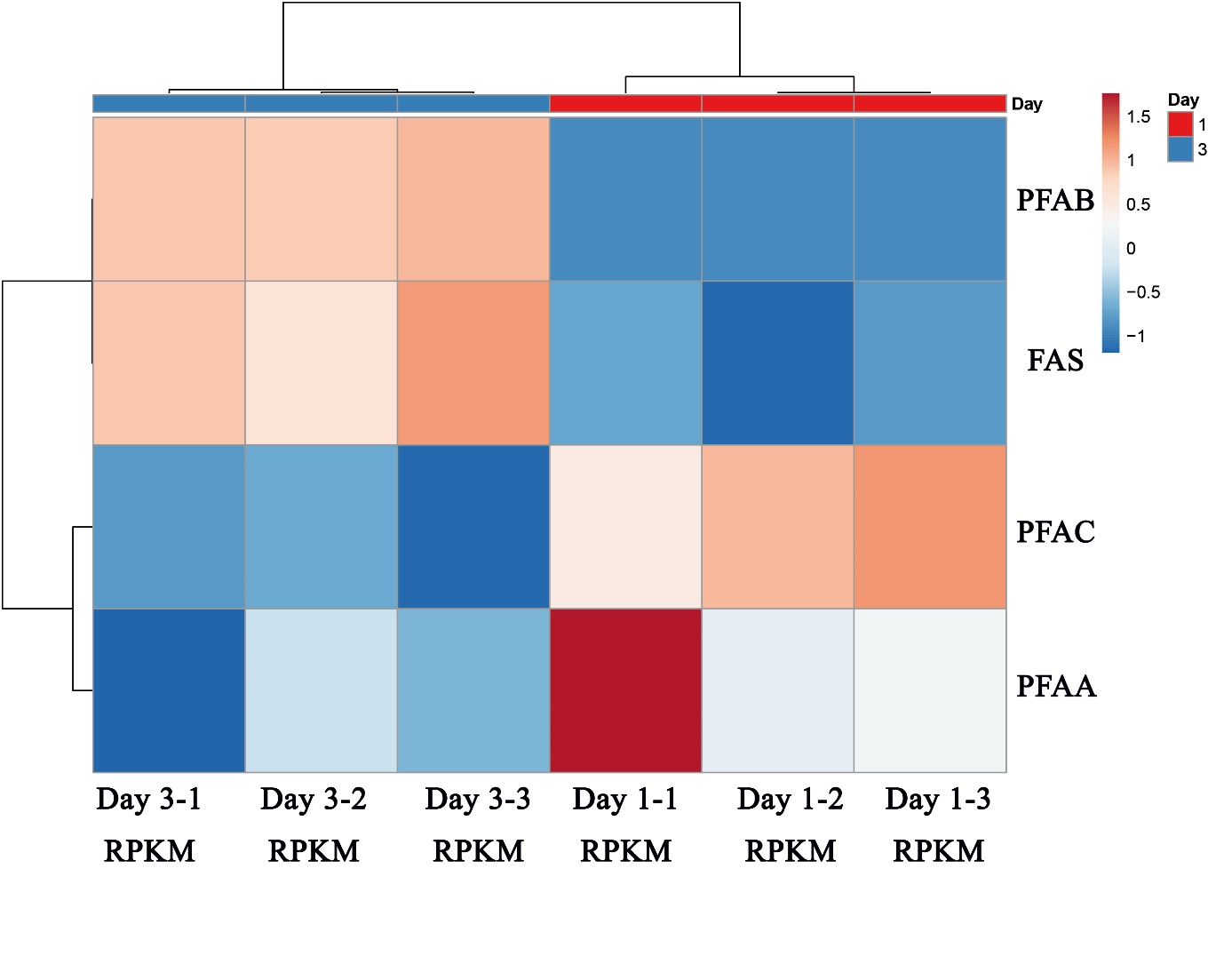


**Figure S11.** Heatmap of differentially expressed *fas, pfaA, pfaB* and *pfaC* genes (FDR p-value<0.05). RPKM values were pre-processed for heatmap generation. Rows were centered, then unit variance scaling was applied to rows. Both rows and columns were clustered using correlation distance and average linkage methods. Rows represent genes (n=4), and columns represent samples (n=6).


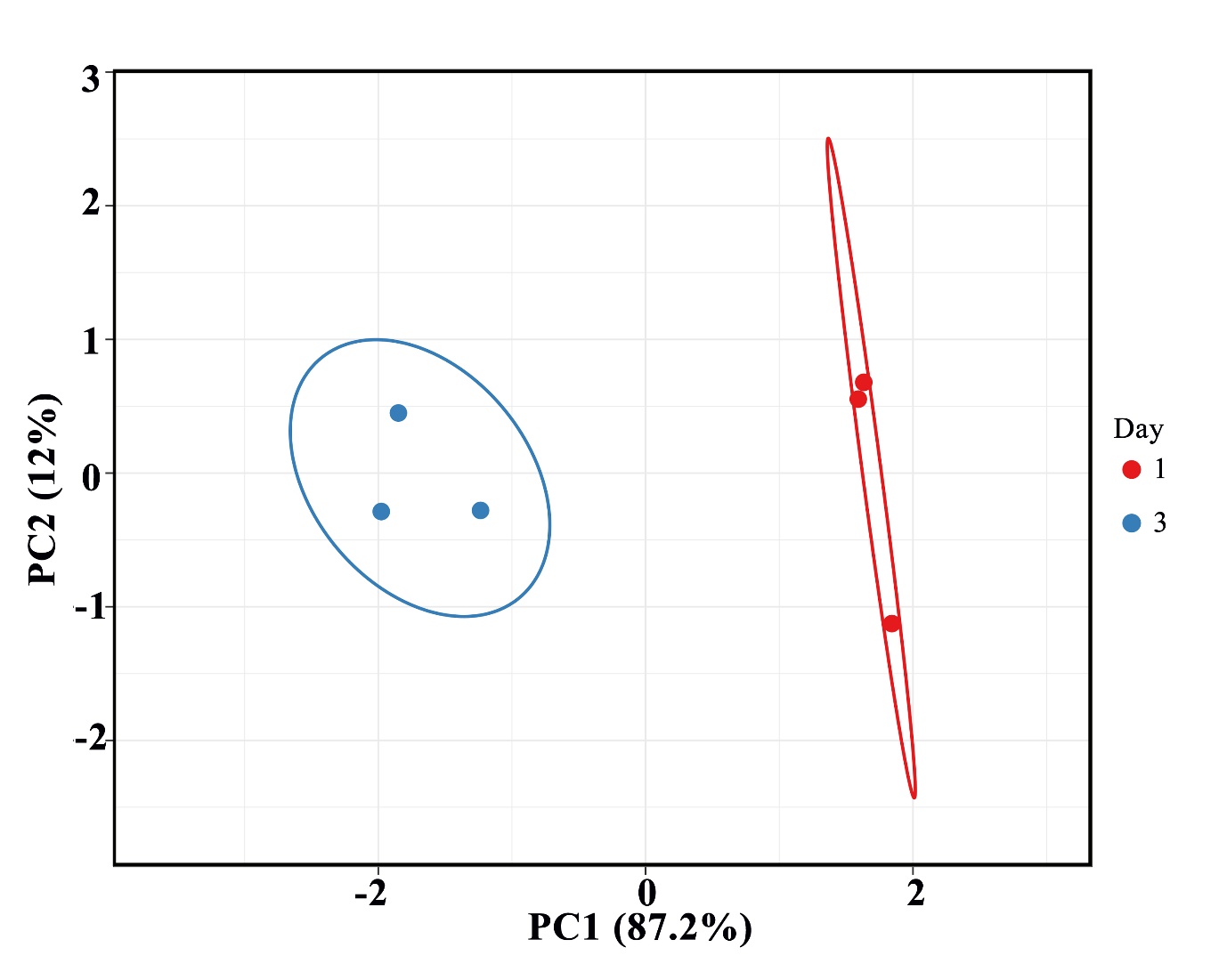


**Figure S12:** Principal component analysis applied to the three replicates for *fas, pfaA, pfaB* and *pfaC* genes (FDR p-value<0.05) associated to lipid metabolism according to the annotation to *Aurantiochytrium* T66 genome.

**Table S3.** KEGG Enrichment analysis of upregulated genes

| **Term ID** | **Term description** | **Observed gene count** |  | **Background gene count** | **False discovery rate** |
| --- | --- | --- | --- | --- | --- |
| hsa01100 | Metabolic pathways | 13 |  | 1250 | 3.19E-07 |
| hsa01200 | Carbon metabolism | 6 |  | 116 | 5.89E-07 |
| hsa01040 | Biosynthesis of unsaturated fatty acids | 4 |  | 23 | 1.30E-06 |
| hsa00030 | Pentose phosphate pathway | 4 |  | 30 | 2.55E-06 |
| hsa01212 | Fatty acid metabolism | 4 |  | 48 | 1.17E-05 |
| hsa00564 | Glycerophospholipid metabolism | 4 |  | 96 | 0.00013 |
| hsa00010 | Glycolysis / Gluconeogenesis | 3 |  | 68 | 0.0014 |
| hsa01230 | Biosynthesis of amino acids | 3 |  | 72 | 0.0014 |
| hsa03320 | PPAR signaling pathway | 3 |  | 72 | 0.0014 |
| hsa00592 | alpha-Linolenic acid metabolism | 2 |  | 25 | 0.0046 |
| hsa00051 | Fructose and mannose metabolism | 2 |  | 33 | 0.007 |
| hsa00071 | Fatty acid degradation | 2 |  | 44 | 0.0111 |

**Table S4**. Reactome Pathways Enrichment analysis of upregulated genes

| **Term ID** | **Term description** | **Observed gene count** | **Background gene count** | **False discovery rate** |
| --- | --- | --- | --- | --- |
| HSA-1430728 | Metabolism | 15 | 2032 | 1.61E-06 |
| HSA-1483206 | Glycerophospholipid biosynthesis | 5 | 128 | 5.10E-05 |
| HSA-556833 | Metabolism of lipids | 8 | 721 | 0.00017 |
| HSA-71387 | Metabolism of carbohydrates | 5 | 266 | 0.00065 |
| HSA-2046105 | Linoleic acid (LA) metabolism | 2 | 8 | 0.0013 |
| HSA-70171 | Glycolysis | 3 | 66 | 0.0016 |
| HSA-2046106 | alpha-linolenic acid (ALA) metabolism | 2 | 12 | 0.002 |
| HSA-71336 | Pentose phosphate pathway | 2 | 15 | 0.0023 |
| HSA-1483191 | Synthesis of PC | 2 | 28 | 0.0061 |
| HSA-70263 | Gluconeogenesis | 2 | 33 | 0.0077 |
| HSA-8978868 | Fatty acid metabolism | 3 | 171 | 0.012 |

*Enriched domains in upregulated genes*

Enriched domains in upregulated genes showed significant enrichment of Fatty acid desaturase, and Cytochrome b5 -like Heme/Steroid binding domain and NAD(P)-binding domain superfamily.

**Table S5**. Top upregulated genes in lipid metabolism category during lipid production process in *Aurantiochytrium* sp. TC20.

| **Name** | **Abbreviation** | **Name** | **Functional group** | **Fold change** | **P-value** | **FDR p-value** |
| --- | --- | --- | --- | --- | --- | --- |
| T66005295.1 | SSDH-2 | Succinate semialdehyde dehydrogenase | Associated reactions, cycles and shunts | 28.48 | 0.00 | 0.00 |
| T66007131.1 | PEPC | Phosphoenolpyruvate carboxylase | Associated reactions, cycles and shunts | 2.17 | 0.00 | 0.00 |
| T66008640.1 | SCCT | Succinyl-CoA D-citramalate CoA-transferase | Associated reactions, cycles and shunts | 2.00 | 0.00 | 0.00 |
| T66008840.1 | AMPD | AMP-deaminase | Associated reactions, cycles and shunts | 1.48 | 0.00 | 0.00 |
| T66005413.1 | PFAB | Polyunsaturated fatty acid synthase subunit B | Fatty acid synthesis | 6.44 | 0.00 | 0.00 |
| T66009075.1 | ACSL-10 | Acyl-CoA fatty acid synthetase long-chain | Fatty acid synthesis | 3.31 | 0.00 | 0.00 |
| T66006792.1 | d5D | Δ5 fatty acid desaturase | Fatty acid synthesis | 2.81 | 0.00 | 0.01 |
| T66005706.1 | ACC | Acetyl-CoA carboxylase | Fatty acid synthesis | 2.81 | 0.00 | 0.00 |
| T66000041.1 | MCAT-3 | Malonyl-CoA acyltransferase putative | Fatty acid synthesis | 2.53 | 0.00 | 0.00 |
| T66000114.1 | ACSL-14 | Acyl-CoA fatty acid synthetase long-chain | Fatty acid synthesis | 2.39 | 0.00 | 0.00 |
| T66000104.1 | ELO-5 | C16-Δ9 elongase | Fatty acid synthesis | 2.34 | 0.00 | 0.00 |
| T66000001.1 | ACSL-1 | Acyl-CoA fatty acid synthetase long-chain | Fatty acid synthesis | 2.31 | 0.00 | 0.00 |
| T66003686.1 | ACSL-5 | Acyl-CoA fatty acid synthetase long-chain | Fatty acid synthesis | 1.96 | 0.00 | 0.00 |
| T66000037.1 | d6D | Δ6 fatty acid desaturase | Fatty acid synthesis | 1.87 | 0.00 | 0.00 |
| T66000055.1 | ACSS-1 | Acyl-CoA fatty acid synthetase short-chain | Fatty acid synthesis | 1.86 | 0.00 | 0.00 |
| T66004341.1 | FATP-2 | Long-chain fatty acid transport protein (with ACSL) | Fatty acid synthesis | 1.85 | 0.00 | 0.00 |
| T66002260.1 | ACOT-2 | Acyl-CoA thioesterase | Fatty acid synthesis | 1.78 | 0.00 | 0.00 |
| T66001622.1 | ACSL-2 | Acyl-CoA fatty acid synthetase long-chain | Fatty acid synthesis | 1.78 | 0.00 | 0.00 |
| T66000039.1 | ACOT-3 | Acyl-CoA thioesterase | Fatty acid synthesis | 1.69 | 0.00 | 0.00 |
| T66000150.1 | PFAD | Phosphopantetheinyl transferase | Fatty acid synthesis | 1.62 | 0.00 | 0.00 |
| T66002859.1 | FATP-1 | Long-chain fatty acid transport protein (with ACSL) | Fatty acid synthesis | 1.60 | 0.00 | 0.00 |
| T66000109.1 | d4D | Δ4 fatty acid desaturase | Fatty acid synthesis | 1.59 | 0.00 | 0.00 |
| T66009076.1 | ACSL-11 | Acyl-CoA fatty acid synthetase long-chain | Fatty acid synthesis | 1.58 | 0.00 | 0.00 |
| T66000040.1 | d12D | Δ12 fatty acid desaturase | Fatty acid synthesis | 1.49 | 0.00 | 0.00 |
| T66000093.1 | ACOT-4 | Acyl-CoA thioesterase | Fatty acid synthesis | 1.42 | 0.00 | 0.00 |
| T66007597.1 | ACSL-9 | Acyl-CoA fatty acid synthetase long-chain | Fatty acid synthesis | 1.42 | 0.00 | 0.00 |
| T66006986.1 | ACSL-7 | Acyl-CoA fatty acid synthetase long-chain | Fatty acid synthesis | 1.39 | 0.00 | 0.00 |
| T66001099.1 | MCAT-2 | Malonyl-CoA acyltransferase putative | Fatty acid synthesis | 1.36 | 0.00 | 0.00 |
| T66007272.1 | ACSL-8 | Acyl-CoA fatty acid synthetase long-chain | Fatty acid synthesis | 1.28 | 0.00 | 0.00 |
| T66004311.1 | FAS | Fatty acid synthase 1 | Fatty acid synthesis | 1.26 | 0.00 | 0.00 |


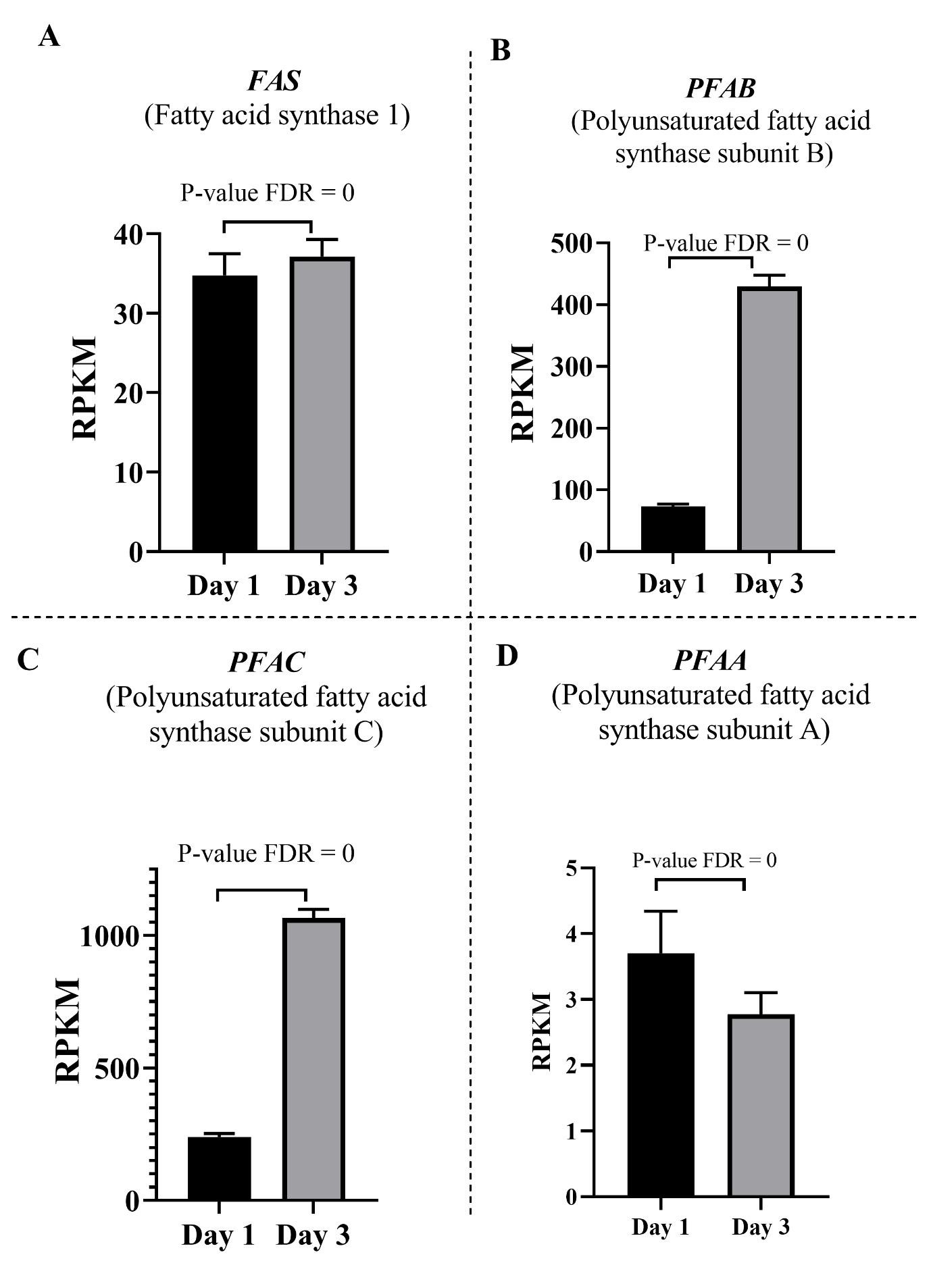


**Figure S13:** Differences in gene expression for A) *fas,* B) *pfaB,* C) *pfaC* and D) *pfaA* genes between days 1 and 3. Values given in RPKM.

**Table S6**. Downregulated genes in lipid metabolism category during lipid production process in *Aurantiochytrium* sp. TC20 with a down regulation over 3 fold.

| **Name** | **Abbreviation** | **Name** | **Functional group** | **Fold change** | **P-value** | **FDR p-value** |
| --- | --- | --- | --- | --- | --- | --- |
| T66004665.1 | GAD | Glutamate decarboxylase | Associated reactions, cycles and shunts | -7.46 | 0.0 | 0.0 |
| T66002600.1 | IDH-1 | Isocitrate dehydrogenase | TCA | -6.46 | 0.0 | 0.0 |
| T66006628.1 | ENO | Enolase | Glycolysis and gluconeogenesis | -6.05 | 0.0 | 0.0 |
| T66001332.1 | AST | Aspartate aminotransferase | Associated reactions, cycles and shunts | -5.03 | 0.0 | 0.0 |
| T66011653.1 | RPI | Ribose-5-phosphate isomerase | PPP | -4.90 | 0.0 | 0.0 |
| T66000362.1 | MDH-1 | Malate dehydrogenase | TCA | -4.85 | 0.0 | 0.0 |
| T66002193.1 | PL | Phospholipase | Lipases | -4.59 | 0.0 | 0.0 |
| T66005762.1 | ACS | Acetyl-CoA synthetase | Associated reactions, cycles and shunts | -4.13 | 0.0 | 0.0 |
| T66010760.1 | SCLB | Succinate - CoA ligase subunit beta | TCA | -3.86 | 0.0 | 0.0 |
| T66006855.2 | PGK-1 | Phosphoglycerate kinase | Glycolysis and gluconeogenesis | -3.79 | 0.0 | 0.0 |
| T66007915.1 | ELO-3 | Fatty acyl-CoA elongase | Fatty acid synthesis | -3.75 | 0.0 | 0.0 |
| T66002129.1 | ICL | Isocitrate lyase | Associated reactions, cycles and shunts | -3.65 | 0.0 | 0.0 |
| T66009538.1 | SDH-4 | Succinate dehydrogenase | TCA | -3.62 | 0.0 | 0.0 |
| T66009371.1 | SDH-3 | Succinate dehydrogenase | TCA | -3.59 | 0.0 | 0.0 |
| T66010787.1 | ACAD | Acyl-CoA dehydrogenase family protein | β-oxidation | -3.57 | 0.0 | 0.0 |
| T66008367.1 | DLDH | Dihydrolipoyl dehydrogenase | Glycolysis and gluconeogenesis | -3.52 | 0.0 | 0.0 |
| T66002966.1 | SDH-1 | Succinate dehydrogenase | TCA | -3.50 | 0.0 | 0.0 |
| T66006417.1 | PAP-3 | Phosphatidic acid phosphatase | TAG-synthesis | -3.47 | 0.0 | 0.0 |
| T66004756.1 | KGMT-2 | α-ketoglutarate/malate carrier protein mitochondria | Transport in/out of mitochondria | -3.32 | 0.0 | 0.0 |
| T66011336.1 | PDHB | Pyruvate dehydrogenase E1 subunit beta | Glycolysis and gluconeogenesis | -3.30 | 0.0 | 0.0 |
| T66005350.1 | FUM-2 | Fumarase | TCA | -3.26 | 0.0 | 0.0 |
| T66007784.1 | ER-3 | Trans-2-enoyl-CoA reductase | Fatty acid synthesis | -3.21 | 0.0 | 0.0 |
| T66003816.1 | HCD | 3-hydroxyacyl-CoA dehydrogenase | β-oxidation | -3.18 | 0.0 | 0.0 |
| T66004470.1 | IDH-2 | Isocitrate dehydrogenase | TCA | -3.12 | 0.0 | 0.0 |
| T66000025.1 | CTP-1 | Citrate transport protein | Transport in/out of mitochondria | -3.09 | 0.0 | 0.0 |
| T66003615.1 | ECH | Enoyl-CoA hydratase domain-containing protein | β-oxidation | -3.04 | 0.0 | 0.0 |
| T66007132.1 | ACAD | Acyl-CoA dehydrogenase family protein | β-oxidation | -3.02 | 0.0 | 0.0 |
| T66005684.1 | GK | Glycerol kinase | Glycerol uptake and conversion to glycerol phosphate | -3.02 | 0.0 | 0.0 |
| T66000024.1 | ACSL-1 | Acyl-CoA fatty acid synthetase long-chain | Fatty acid synthesis | -2.92 | 0.0 | 0.0 |
| T66007922.1 | ELO-4 | Fatty acyl-CoA elongase | Fatty acid synthesis | -2.89 | 0.0 | 0.0 |
| T66011482.1 | TKT | Transketolase | PPP | -2.88 | 0.0 | 0.0 |
| T66006276.1 | FRD-2 | Fumarate reductase | Associated reactions, cycles and shunts | -2.78 | 0.0 | 0.0 |
| T66001838.1 | GUP-1 | Glycerol uptake facilitator protein | Glycerol uptake and conversion to glycerol phosphate | -2.77 | 0.0 | 0.0 |
| T66007895.1 | ME-2 | Malic enzyme | Associated reactions, cycles and shunts | -2.66 | 0.0 | 0.0 |
| T66010974.1 | PGK-2 | Phosphoglycerate kinase | Glycolysis and gluconeogenesis | -2.61 | 0.0 | 0.0 |
| T66001706.1 | DGK | Diacylglycerol kinase | PL-synthesis | -2.57 | 0.0 | 0.0 |
| T66008195.1 | PSS | Phosphatidylserine decarboxylase | PL-synthesis | -2.53 | 0.0 | 0.0 |
| T66010775.1 | GAPDH-1 | Glyceraldehyde-3-phosphate dehydrogenase | Glycolysis and gluconeogenesis | -2.39 | 0.0 | 0.0 |
| T66011516.1 | TAL | Transaldolase | PPP | -2.38 | 0.0 | 0.0 |
| T66004341.1 | FATP-2 | Long-chain fatty acid transport protein (with ACSL) | Fatty acid synthesis | -2.36 | 0.0 | 0.0 |
| T66006817.1 | SCLA | Succinate - CoA ligase subunit alpha | TCA | -2.36 | 0.0 | 0.0 |
| T66010794.1 | AKGDH-3 | Alpha-ketoglutarate dehydrogenase | TCA | -2.34 | 0.0 | 0.0 |
| T66006290.1 | ER-1 | Trans-acting enoyl reductase | Fatty acid synthesis | -2.32 | 0.0 | 0.0 |
| T66002465.1 | GUP-2 | Glycerol uptake facilitator protein | Glycerol uptake and conversion to glycerol phosphate | -2.28 | 0.0 | 0.0 |
| T66001999.1 | PGL | 6-phosphogluconolactonase | PPP | -2.26 | 0.0 | 0.0 |
| T66008352.1 | ECI | Enoyl-CoA delta isomerase | β-oxidation | -2.23 | 0.0 | 0.0 |
| T66006246.1 | d8D | Δ8 fatty-acid desaturase | Fatty acid synthesis | -2.20 | 0.0 | 0.0 |
| T66002738.1 | ACSL-3 | Acyl-CoA fatty acid synthetase long-chain | Fatty acid synthesis | -2.19 | 0.0 | 0.0 |
| T66002260.1 | ACOT-2 | Acyl-CoA thioesterase | Fatty acid synthesis | -2.15 | 0.0 | 0.0 |
| T66005536.1 | SDH-2 | Succinate dehydrogenase | TCA | -2.14 | 0.0 | 0.0 |
| T66005296.1 | ECHP | Bifunctional enzyme (Enoyl-CoA hydratase/3,2-trans-enoyl-CoA isomerase) | β-oxidation | -2.14 | 0.0 | 0.0 |
| T66011598.1 | CS | Citrate synthase | TCA | -2.13 | 0.0 | 0.0 |
| T66003371.1 | HADHB | Hydroxyacyl-CoA dehydrogenase trifunctional | β-oxidation | -2.10 | 0.0 | 0.0 |
| T66011468.1 | CCP | Citrate/ketoglutarate carrier protein | Transport in/out of mitochondria | -2.09 | 0.0 | 0.0 |
| T66002557.1 | AST | Aspartate aminotransferase | Associated reactions, cycles and shunts | -2.04 | 0.0 | 0.0 |
| T66004579.1 | ACSS-1 | Acyl-CoA fatty acid synthetase short-chain | Fatty acid synthesis | -2.04 | 0.0 | 0.0 |
| T66006070.1 | ME-1 | Malic enzyme | Associated reactions, cycles and shunts | -2.02 | 0.0 | 0.0 |
| T66006391.1 | MGL | Monoacylglycerol lipase | Lipases | -2.01 | 0.0 | 0.0 |
|  |  |  |  |  |  |  |
